# Construction of a MOF‐Based Snap‐Top Delivery Nanosystem for Powerful Dual‐Responsive Synergistic Colitis Treatment

**DOI:** 10.1002/advs.202524174

**Published:** 2026-04-02

**Authors:** Xin Li, Miao Xu, Heran Li, Huan Jiang, Xin Wang, Lianjun Ma, Ying‐Wei Yang

**Affiliations:** ^1^ International Joint Research Laboratory of Nano‐Micro Architecture Chemistry College of Chemistry Jilin University Changchun China; ^2^ School of Pharmacy China Medical University Shenyang China; ^3^ Jilin Provincial Key Laboratory of Tooth Development and Bone Remodeling Department of Orthodontics School and Hospital of Stomatology Jilin University Changchun China; ^4^ China‐Japan Union Hospital Jilin University Changchun China

**Keywords:** drug delivery, metal–organic frameworks, responsive materials, supramolecular materials, synergistic therapy

## Abstract

Oral treatment of inflammatory bowel disease (IBD) faces significant challenges of poor targeting, low bioavailability, and systemic toxicity, necessitating intelligent stimuli‐responsive delivery systems. Here, we design and synthesize a pH/hypoxia dual‐responsive pendant azobenzene‐functionalized MOF (Azo‐MOF). The uniformly distributed azobenzene pendants serve as intrinsic gating sites, forming “snap‐top” encapsulation with β‐cyclodextrin through host‐guest interactions that prevent premature 6‐mercaptopurine leakage while enabling rapid stimuli‐triggered release, creating a postmodification‐free supramolecular gated delivery nanosystem (CAMM). CAMM exhibits hypoxia‐triggered burst release and alkaline‐sustained release, with <5.0% drug leakage during gastrointestinal transit and >90.0% cumulative release at inflammatory sites. Notably, the Azo‐MOF carrier scavenges reactive oxygen species through synergy between its conjugated *π*‐system and metal coordination environment, providing antioxidant protection alongside drug delivery. In dextran sulfate sodium‐induced colitis models, CAMM outperformed free drug, achieving near‐complete weight recovery, downregulating proinflammatory cytokines, restoring intestinal barrier integrity, and rebalancing gut microbiota by enriching *Lactobacillus* and *Bifidobacterium* while suppressing opportunistic pathogens. This study integrates targeted delivery, antioxidant therapy, and microbiota modulation, offering an effective strategy for precision IBD treatment.

## Graphical abstract

A snap‐top delivery nanosystem based on an azobenzene metal–organic framework achieves targeted release of 6‐mercaptopurine via acid‐stable frameworks and hypoxia/pH dual‐responsive β‐cyclodextrin gates. This multifunctional platform integrates three synergistic mechanisms: precision drug delivery to inflammatory sites, intrinsic antioxidant properties that alleviate oxidative stress, and gut microbiota modulation that enriches beneficial *Lactobacillus* while suppressing pathogenic *Enterococcus*, thereby comprehensively restoring intestinal homeostasis in colitis.

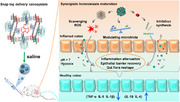

## 1 Introduction

Oral drug delivery for inflammatory bowel disease (IBD) faces substantial challenges from the complex pathophysiological environment of the inflamed intestinal tract [1]. Current therapeutic approaches exemplified by immunomodulatory agents such as 6‐mercaptopurine (6‐MP) illustrate this clinical dilemma. While 6‐MP possesses the potent anti‐inflammatory and immunosuppressive activities essential for long‐term IBD therapy [2], oral administration results in rapid enzymatic degradation by xanthine oxidase, yielding inactive metabolites with only minimal conversion to therapeutically active 6‐thioguanine nucleotides (6‐TGN) [3]. This low bioavailability necessitates high‐dose regimens, leading to toxic metabolite accumulation that causes adverse reactions, including hepatotoxicity and myelosuppression in approximately 25% of patients [4, 5, 6]. The resulting efficacy‐toxicity paradox constrains the clinical utility of 6‐MP, highlighting the urgent need for smart delivery systems.

Oral drug delivery systems have evolved from conventional formulations [7, 8] to delivery platforms utilizing liposomes [9, 10, 11], polymers [12, 13], inorganic nanoparticles [14], framework materials [15, 16, 17, 18, 19], and other advanced nanocarriers [20, 21, 22, 23, 24], however, achieving precision targeting and controlled release remains challenging. Supramolecular gating systems provide a new solution through stimulus‐responsive molecular valves that enable spatiotemporal control of drug release [25]. The snap‐top concept pioneered by Stoddart and Zink achieves rapid pore opening of silica nanocontainers through stimulus‐triggered dissociation of molecular valves [26]. Building upon this groundbreaking strategy, researchers have extended the snap‐top mechanism from silica‐based materials to diverse nanoplatforms to achieve tissue‐specific targeting and multi‐stimuli responsiveness [27, 28, 29, 30]. Metal–organic frameworks (MOFs) have emerged as star carriers in gated delivery systems due to their high surface area, tunable pore structures, and good biocompatibility [31]. Our group pioneered the design and construction of MOF‐based supramolecular gating platforms [32, 33], yet conventional gating assemblies relying on post‐synthetic modification suffer from inhomogeneous functionalization and structural instability, resulting in premature drug leakage and off‐target risks. To address these limitations, we developed an in situ ligand functionalization strategy to synthesize a pendant azobenzene‐functionalized MOF (Azo‐MOF) with stimuli‐responsive gating sites directly integrated into the framework, eliminating postmodification steps while enhancing response precision for efficient IBD therapy.

**FIGURE 1:**
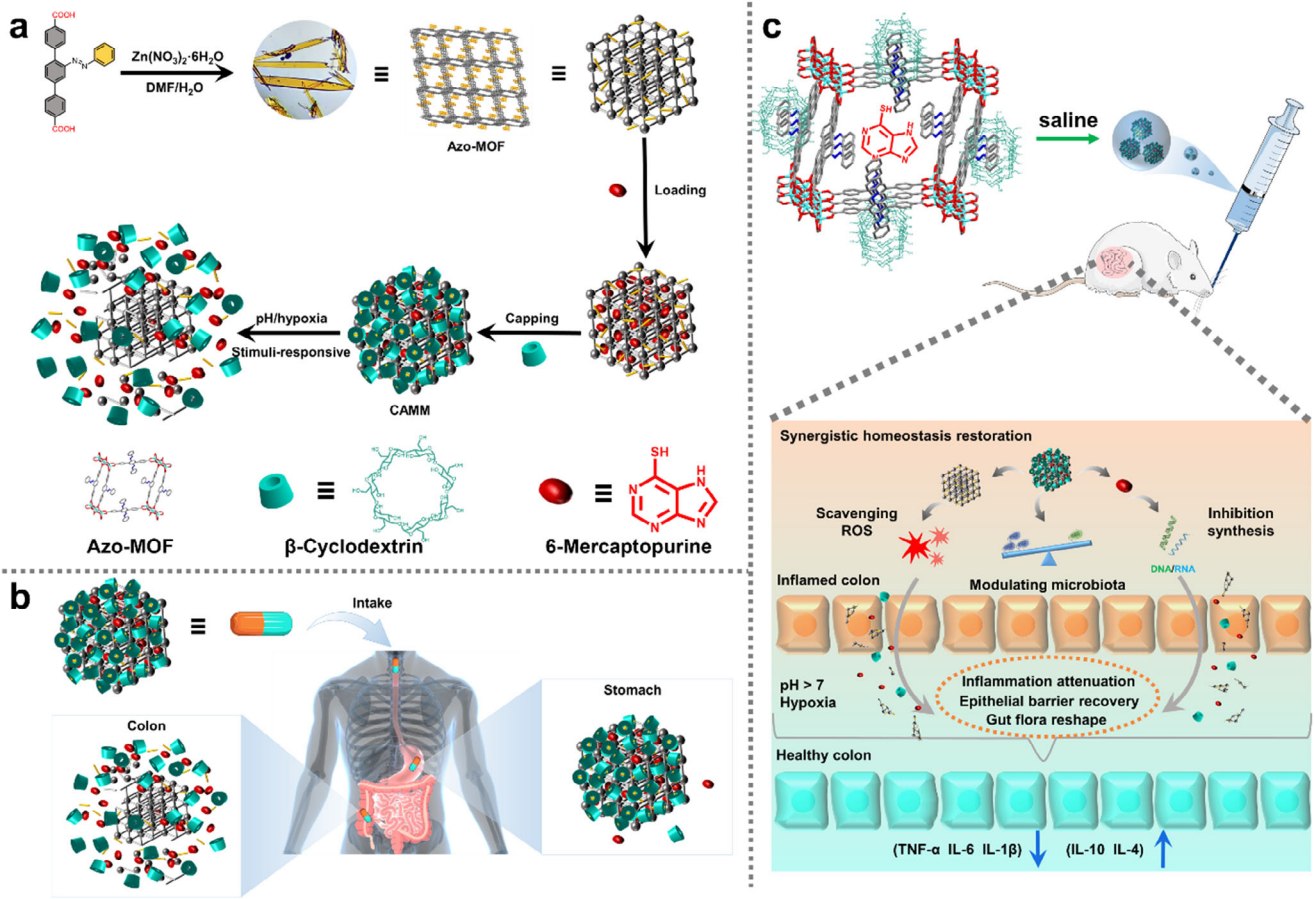
Design, fabrication, and therapeutic mechanism of the CAMM delivery system. (a) Synthesis of Azo‐MOF and construction of a supramolecular gated delivery system loaded with 6‐MP, β‐CD‐capped with dual‐stimuli responsive properties (azobenzene units as stalks encircled by β‐CD on the surface). (b) Schematic illustration of targeted drug release after gastrointestinal transit of the delivery system. (c) Therapeutic mechanism of CAMM against DSS‐induced colitis in mice.

Based on this design, we constructed a “snap‐top” delivery nanosystem by loading 6‐MP into Azo‐MOF and capping with biocompatible β‐cyclodextrin (β‐CD), yielding CAMM (abbreviated from β‐Cyclodextrin‐capped Azo‐MOF‐loaded 6‐Mercaptopurine) for multi‐mechanism synergistic IBD therapy (Figure 1a). The azobenzene pendants serve as efficient host‐guest recognition sites, forming stable supramolecular gates with β‐CD that effectively prevent premature drug leakage [34, 35]. Azobenzene bonds are cleavable by azoreductase under hypoxic inflammatory conditions, opening the molecular cap and triggering precise drug release [36, 37, 38]. The π‐conjugated systems synergize with metal‐ligand networks to enhance gastric acid stability [39], while alkaline intestinal conditions can promote further MOF degradation and drug release [40]. This hypoxia/pH dual‐responsive mechanism achieves targeted 6‐MP delivery at inflammatory sites (Figure 1b). In dextran sulfate sodium (DSS)‐induced colitis mouse models, CAMM demonstrated effective therapeutic outcomes, encompassing intestinal epithelial tissue recovery, reduced inflammatory cytokine levels, and restored barrier function, while minimizing systemic toxicities associated with 6‐MP monotherapy. Notably, we discovered that the Azo‐MOF carrier itself possesses potent reactive oxygen species (ROS) scavenging capability, thereby creating synergistic therapeutic effects with 6‐MP immunomodulatory action. Furthermore, the CAMM system effectively modulates intestinal microbiome composition and restores bacterial homeostasis, achieving a transition from sole drug delivery toward comprehensive microenvironmental regulation (Figure 1c).

## 2 Results

### 2.1 Design and Construction of the Snap‐Top Delivery Nanosystem

Traditional supramolecular gated systems require post‐synthetic modification to introduce functional linkers (such as quaternary ammonium salts or pyridine moieties) onto carrier surfaces as anchor sites for valve attachment [41, 42, 43]. However, this process involves complex operations, low modification efficiency, and an uneven distribution, ultimately compromising structural integrity and impairing delivery performance. Therefore, we designed an Azo‐MOF with the formula [Zn_3_(*L*)_3_(µ_2_‐O)(µ_3_‐O)] using 2'‐phenyldiazenyl‐1,1':4',1″‐terphenyl‐4,4″‐dicarboxylate ligands (Azo‐ligand, Figures S1–S4) and biocompatible Zn^2+^ [44]. The inherent azobenzene pendants serve directly as linkers, binding to β‐CD via host‐guest interactions to efficiently cap 6‐MP‐loaded MOF channels.

**FIGURE 2:**
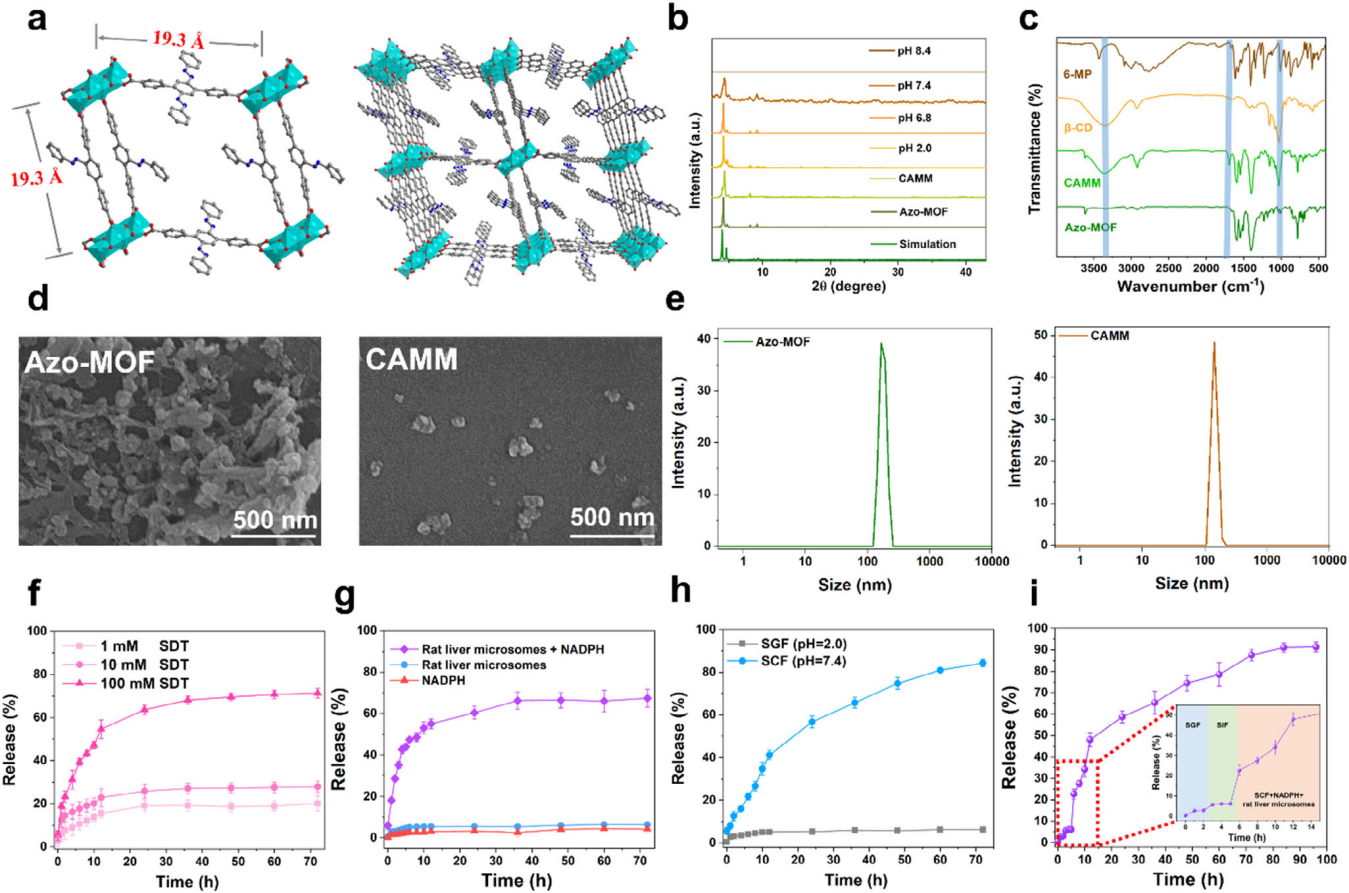
Crystal structure of Azo‐MOF and stimuli‐responsive release kinetics of CAMM. (a) Crystal structure of Azo‐MOF. (b) PXRD patterns of simulated Azo‐MOF, as‐synthesized Azo‐MOF, CAMM, and Azo‐MOF treated with buffer solutions at different pH values. (c) FT‐IR spectra of CAMM and its individual components (6‐MP, β‐CD, Azo‐MOF). (d) SEM images of Azo‐MOF and CAMM. (e) Particle size distribution profiles of Azo‐MOF and CAMM. (f) Release kinetics of CAMM in PBS buffer under hypoxic conditions with different SDT concentrations (1, 10, and 100 mM). (g) Drug release profiles of CAMM in PBS buffer (pH 6.8) containing rat liver microsomes supplemented with nicotinamide adenine dinucleotide phosphate (NADPH), compared with rat liver microsomes alone and NADPH alone as controls. (h) Release kinetics of 6‐MP from CAMM in simulated gastric fluid (SGF, pH 2.0) and simulated colonic fluid (SCF, pH 7.4). (i) Drug release of CAMM during simulated gastrointestinal transit in colitis conditions, sequentially passing through SGF, simulated intestinal fluid (SIF, pH 6.8), and SCF (containing rat liver microsomes and NADPH).

Single‐crystal analysis revealed a uniform distribution of azobenzene pendants along square channels (Figure 2a; Figure S5, Table S1). The 19.3 Å × 19.3 Å channel dimensions provide ample drug loading space, while BET surface area analysis yielded 594 m^2^ g^−1^ (Figures S6 and S7). The TGA analysis reveals that the Azo‐MOF framework remains thermally stable up to 425°C (Figure S8). To evaluate capping performance, we compared drug release profiles between uncapped and capped carriers using UV–Vis spectroscopy. Uncapped carriers showed continuous burst release of drug, while capped carriers exhibited virtually no detectable leakage with cumulative release rates below 5.0% (Figure S9). N_2_ sorption isotherms of capped carriers showed non‐porous material characteristics with significantly reduced surface area, providing structural evidence for efficient capping (Figures S6 and S7).

The framework retained its structural integrity during drug loading, as evidenced by PXRD analysis (Figure 2b). Meanwhile, pH‐stability studies revealed strong stability at pH 2, framework collapse at pH > 7, and complete disintegration at pH > 8, enabling targeted colonic delivery. SEM imaging further confirmed that CAMM retained its morphology at pH 2.0, whereas it exhibited significant structural disintegration at pH 7.4 (Figure S10), corroborating the pH‐responsive degradation behavior. FT‐IR spectroscopy confirmed successful CAMM composite construction (Figure 2c) with characteristic peaks at 1690 cm^−1^ (6‐MP C═N stretching), 3357 cm^−1^, and 1024 cm^−1^ (β‐CD O─H and C─O stretching). Drug loading and β‐CD capping installation increased zeta potential by 15.8 mV compared to pristine Azo‐MOF, indicating enhanced colloidal stability (Figure S11). SEM analysis revealed improved CAMM dispersion compared to Azo‐MOF (Figure 2d), attributed to surface‐bound hydrophilic cyclodextrin, which improved aqueous dispersibility. At the same time, DLS measurements showed a 22.4 nm reduction in average particle size (Figure 2e). These results demonstrate the successful assembly of a structurally stable snap‐top delivery platform.

### 2.2 Hypoxia/pH Dual‐Responsive Drug Release

In hypoxic, inflammatory environments, elevated azoreductase levels facilitate cleavage of azo bonds [45]. To validate this hypoxia‐responsive behavior, we initially employed sodium dithionite (SDT) as a chemical reductant under hypoxic conditions to mimic the reductive activity of azoreductase. In PBS buffer (pH 6.8), CAMM exhibited concentration‐dependent release kinetics across varying SDT concentrations (1, 10, and 100 mM) (Figure 2f), preliminarily confirming the responsiveness of azo bonds to hypoxic reductive environments. The responsive mechanism was further validated using rat liver microsomes as a biological reductase system (Figure 2g). Liver microsomes are enriched with diverse reductase enzymes that, in the presence of the cofactor nicotinamide adenine dinucleotide phosphate (NADPH), catalyze the reductive cleavage of azo bonds. In PBS buffer (pH 6.8) containing rat liver microsomes supplemented with NADPH, CAMM achieved a release rate of 10.7%/h during the initial 4 h with a cumulative release of 42.7%, reaching near‐saturation (67.3%) within 36 h. Compared with controls using microsomes alone or NADPH alone, this rapid burst release exemplifies the snap‐top hypoxia‐responsive mechanism, wherein enzymatic cleavage of azo bonds rapidly opens the β‐CD cap, enabling immediate drug liberation.

pH‐responsive release studies were conducted in simulated gastric fluid (SGF, pH 2.0) and simulated colonic fluid (SCF, pH 7.4) (Figure 2h). Under acidic gastric conditions, drug leakage remained below 5.0%, ensuring CAMM stability in the gastric acid environment. In alkaline colonic fluid (SCF, pH 7.4), the gradual degradation of the MOF framework triggered a sustained release, with an initial rate of 4.0%/h, achieving 16.0% cumulative release at 4 h and 84.3% at 72 h. Notably, the hypoxia‐responsive release rate during the first 4 h was 2.7‐fold higher than the pH‐responsive rate, while the maximum pH‐responsive release was 1.25‐fold greater than that under hypoxia. This disparity reveals the synergistic interplay of the dual‐stimuli mechanism, in which hypoxia‐triggered burst release ensures immediate drug action at inflammatory sites. In contrast, pH‐mediated sustained release prolongs therapeutic duration, together achieving efficient and precise drug delivery.

This dual‐responsive behavior was further validated in simulated gastrointestinal transit under colitis conditions (Figure 2i). Sequential passage through SGF and simulated intestinal fluid (SIF, pH 6.8) resulted in less than 5.0% drug leakage, effectively preventing premature release in the upper gastrointestinal tract. Upon reaching the simulated inflammatory site (SCF containing rat liver microsomes and NADPH), dual stimulation by hypoxic and alkaline microenvironments triggered a rapid release of 48.0% of the drug, followed by a sustained release extending to 96 h, with a maximum cumulative release of 91.3%.

**FIGURE 3:**
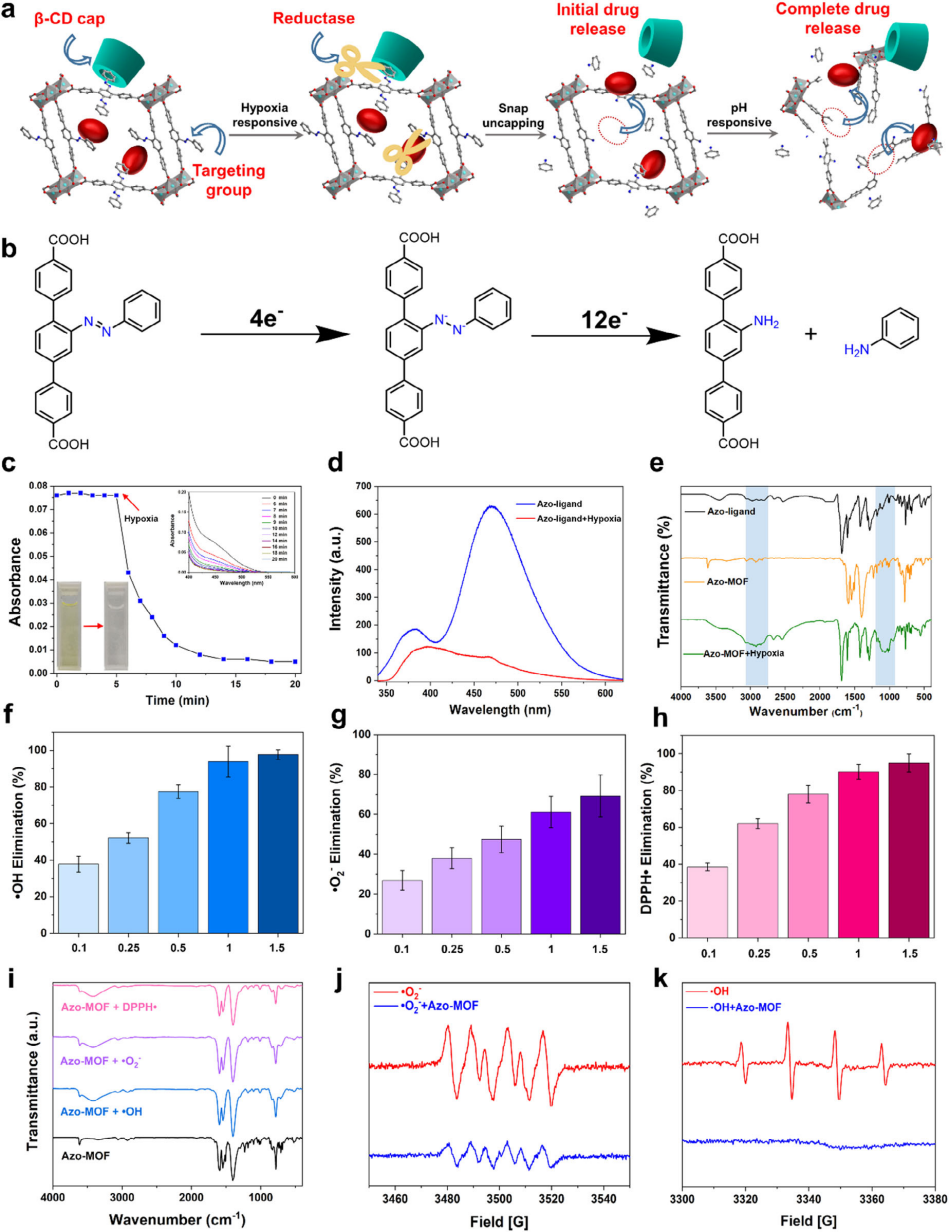
Snap‐top release mechanism of CAMM and radical scavenging capacity of Azo‐MOF. (a) Schematic illustration of the dual‐responsive drug release mechanism. (b) Chemical reaction scheme of Azo‐ligand cleavage process from CAMM under azoreductase catalysis. (c) Absorbance at 450 nm of Azo‐ligand (10 µM) in PBS buffer (pH 6.8) containing rat liver microsomes (3.0 mg mL^−1^) and NADPH (40 µM) under hypoxic conditions. Inset: UV–Vis absorption spectra and photographs before and after enzymatic reduction. (d) Fluorescence spectra of Azo‐ligand before and after treatment with rat liver microsomes and NADPH under hypoxic conditions. (e) FTIR spectra of Azo‐MOF before and after treatment with rat liver microsomes and NADPH under hypoxic conditions. Concentration‐dependent radical scavenging activity of Azo‐MOF against •OH (f), •O_2_^−^ (g), and DPPH• (h). Data are presented as means ± SD, *n *= 3. (i) FTIR spectra demonstrating structural stability of Azo‐MOF after ROS scavenging reactions. Electron paramagnetic resonance (EPR) spectra of Azo‐MOF before and after superoxide (j) and hydroxyl radical (k) scavenging, respectively. Source data are provided in the Source Data file.

CAMM responds to hypoxic inflammatory conditions and alkaline microenvironments through complementary molecular mechanisms. Under hypoxic conditions, enzymatic reductive cleavage of azo bonds first opens the β‐CD cap, triggering rapid burst drug release. Subsequently, the alkaline microenvironment induces gradual degradation of the MOF framework, further promoting sustained drug release. This “cap‐opening followed by framework‐degradation” stepwise release mechanism ensures precise spatiotemporal drug delivery at inflammatory sites (Figure 3a). pH responsiveness arises from MOF framework instability under alkaline conditions (Figure 2b), while hypoxia responsiveness involves multi‐electron reduction processes that convert azobenzene functionalities into aromatic amine derivatives (Figure 3b).

To validate the reductive cleavage of azo bonds under hypoxic conditions, we monitored the degradation behavior of Azo‐ligand (10 µM) in PBS buffer (pH 6.8) containing rat liver microsomes and NADPH. The degradation process was tracked through absorbance decay at 450 nm (Figure 3c), with UV–Vis spectral evolution and color transitions confirming complete azo reduction. In the presence of rat liver microsomes and NADPH, complete elimination of the azobenzene‐characteristic emission at 475 nm (Figure 3d) directly established azo bond cleavage. Infrared spectroscopic analysis further validated this transformation, where absorption peaks near 1000 cm^−1^ correspond to aromatic C─N stretching modes of the reduction products, while the broad absorption at 3000 cm^−1^ is attributed to N‐H stretching vibrations of aromatic amines (Figure 3e), confirming the successful conversion of azobenzene functionalities into aromatic amine derivatives. These characterization results collectively verify that CAMM achieves dual‐stimuli‐responsive, precise drug delivery through hypoxia‐triggered cap opening and pH‐mediated framework degradation.

### 2.3 Antioxidant Activity of Azo‐MOF

IBD pathogenesis involves heightened oxidative stress with significantly elevated ROS levels at inflammatory sites. The Azo‐MOF architecture features multiple electron‐rich sites conducive to radical scavenging via conjugated *π*‐electron systems within azo linkages (─N═N‐) and electron‐dense environments surrounding zinc coordination centers. These structural features position the material as an effective electron donor toward electron‐deficient radical species. Antioxidant capacity was tested by scavenging multiple ROS, including hydroxyl radicals, superoxide anions, and DPPH radicals. Quantitative analysis revealed concentration‐dependent scavenging activities of Azo‐MOF, achieving clearance rates of 96.0%, 69.0%, and 95.0% for hydroxyl radicals (•OH), superoxide anions (•O_2_^−^), and DPPH• radicals, respectively, at 1.5 mg mL^−1^ (Figure 3f–h).

Fourier‐transform infrared (FTIR) spectroscopy demonstrated minimal framework perturbation following radical exposure, indicating that scavenging proceeds through physical interactions rather than structural degradation (Figure 3i). Proposed interaction sites include lone‐pair electrons on azo nitrogen atoms, electron‐rich regions of carboxylate ligands, and π–π interaction domains within MOF channels. Electron paramagnetic resonance (EPR) spectroscopy confirmed the radical elimination capacity of Azo‐MOF in xanthine/xanthine oxidase and Fenton reaction systems. Near‐complete EPR signal suppression verified effective scavenging of •OH and •O_2_^−^ species (Figure 3j,k). To validate cellular‐level antioxidant activity, intracellular ROS regulation was assessed in LPS‐activated RAW 264.7 macrophages using the DCFH‐DA fluorescent probe. Azo‐MOF treatment exhibited concentration‐dependent ROS scavenging, with increasing doses (25, 50, 100 µg mL^−1^) progressively reducing intracellular ROS fluorescence intensity compared to LPS‐only controls (Figure S12), confirming its capacity to mitigate inflammatory oxidative stress in activated macrophages.

### 2.4 Targeted Accumulation and Mucosal Penetration in the Inflamed Colon

The mucus barrier presents formidable challenges for drug delivery to inflamed colonic tissues. This viscoelastic network of highly glycosylated mucins covers the intestinal epithelial surface, maintaining intestinal homeostasis while impeding the penetration of therapeutic molecules. In IBD, the mucus barrier undergoes significant pathological remodeling. Enhanced mucin secretion and abnormal crosslinking may strengthen local mucus layer resistance, yet inflammation‐mediated epithelial damage, ulcer formation, and mucus layer discontinuity create potential “windows” for drug penetration. This paradoxical dual effect creates spatially heterogeneous penetration barriers in inflammatory environments, featuring both locally enhanced mucus resistance and pathological penetration channels. Drug delivery systems with excellent mucosal adhesion and penetration properties are therefore critical for achieving precise delivery and sustained residence in complex inflammatory microenvironments, thereby improving therapeutic outcomes.

**FIGURE 4:**
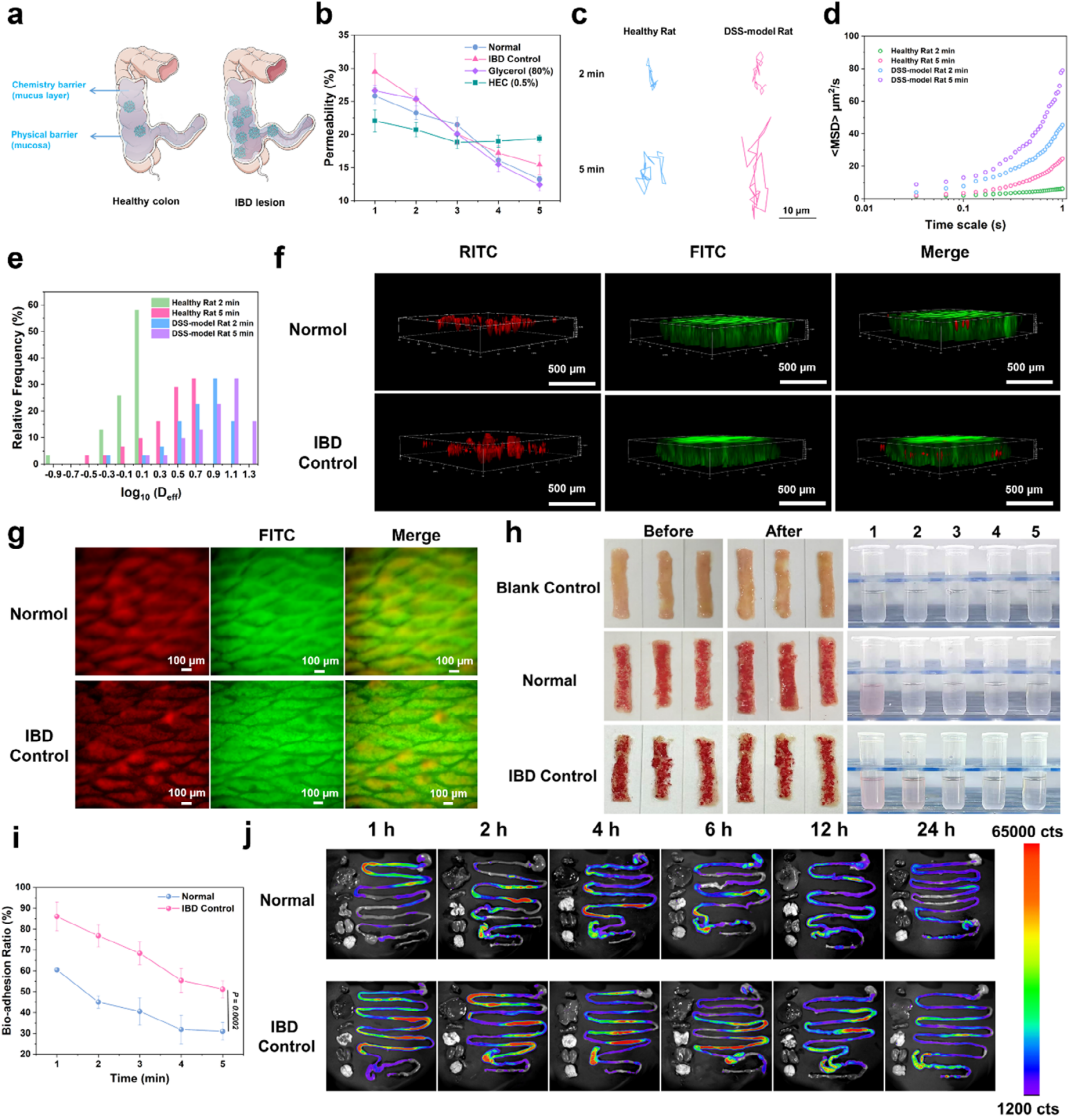
Mucus penetration and biodistribution of rhodamine isothiocyanate (RITC)‐labeled CAMM (CAMMR) nanoparticles. (a) Schematic of nanoparticle penetration in colon mucus. (b) Permeability of CAMMR in rat mucus, glycerol, and crosslinked hydroxyethyl cellulose (HEC). Data are presented as means ± standard deviation, *n* = 3. (c) Representative trajectories of nanoparticle multiple particle tracking in mucus from healthy rats and DSS model rats. (d) Mean square displacement (MSD) vs. time curves. (e) Logarithmic distribution histogram of effective diffusion coefficient (D_eff_)_._ At least 100 particles of each type were tracked for each sample. (f) 3D penetration of CAMMR in rat mucus. Green: mucus stained with fluorescein isothiocyanate (FITC). Red: nanocarriers stained with RITC, scale bar: 500 µm. (g) 2D coverage of CAMMR in rat mucus. Green: mucus stained with FITC. Red: nanocarriers stained with RITC, scale bar: 100 µm. (h) Adhesion status of CAMMR nanoparticles on rat intestinal mucosa and residual conditions after elution. The experiment was independently repeated three times with similar results. (i) Adhesion rate of CAMMR on rat mucosa after 5 min elution. Data are presented as means ± SD, *n* = 3. (j) Fluorescence distribution images of CAMMR in the gastrointestinal tract of normal rats and IBD control rats at 1–24 h after oral administration, and biodistribution in major organs (heart, liver, spleen, lung, kidney, brain). The experiment was independently repeated three times with similar results. The color scale indicates fluorescence intensity. Source data are provided in the Source Data file.

CAMM gastrointestinal retention and lesion localization were investigated (Figure 4a). For ex vivo and in vivo tracking, rhodamine isothiocyanate (RITC) carboxyl groups coordinated to exposed metal sites in CAMM, yielding fluorescently labeled CAMM (CAMMR) with fluorescence stability (Figures S13 and S14). Viscous glycerol (Gly), crosslinked hydroxyethyl cellulose (HEC), and rat‐derived mucus were used to simulate physiological and inflammatory mucus factors, including viscosity and spatial constraints. The CAMMR was placed on 2 cm thick medium surfaces, and penetration behavior was determined over 24 h of free diffusion (Figure S15). CAMMR penetration efficiency in IBD rat intestinal mucus significantly exceeded that of healthy controls (Figure 4b), indicating that inflammatory microenvironments provide favorable conditions for nanocarrier penetration.

Multiple‐particle tracking technology analyzed particle trajectories, diffusion rates, and diffusion efficiency. Particle trajectory visualization showed that CAMMR in the DSS model rat mucus exhibited enhanced linear displacement and diffusion radii, displaying more free movement patterns (Figure 4c). Mean square displacement (MSD) time‐dependence analysis revealed individual particle MSD values in DSS‐model mucus increased 7.2‐fold compared to healthy controls within 5‐min observation windows, with diffusion behavior transitioning from typical restricted diffusion toward near‐Brownian motion (Figure 4d). This transition reflects loosening of the microstructural mucus network. Effective diffusion coefficient (D_eff_) distribution spectral analysis revealed mucus penetration heterogeneity characteristics in inflammatory microenvironments. DSS‐model mucus D_eff_ values showed order‐of‐magnitude increases compared with healthy controls, with broader distribution ranges (Figure 4e). This distribution heterogeneity expansion reflects the spatial non‐uniformity of intestinal mucus microenvironments in inflammatory states.

Fresh ex vivo rat intestinal tube models were used to comprehensively examine CAMMR mucus penetration capacity in pathological states. Fluorescein isothiocyanate (FITC)‐labeled mucus layers were monitored in real‐time using confocal laser scanning microscopy (CLSM) (Figure 4f, g). Z‐axis stacking imaging showed CAMMR exhibited a stronger fluorescence signal intensity and deeper penetration depths in the IBD model rat intestinal tubes. Quantitative analysis revealed CAMMR penetration depths of 201.0 µm and 254.1 µm in healthy and IBD rat intestinal mucus, respectively, representing a 26.4% improvement (Figure S16). These results align with our previous in vitro mucus‐penetration experiments, further confirming CAMMR's adaptability to IBD microenvironments.

Dynamic tracking experiments revealed CAMMR kinetic characteristics in different pathological states. Two‐minute and 5‐min real‐time imaging showed that CAMMR Brownian motion velocity in IBD rat mucus exceeded that of healthy controls (Videos S1–S4). These velocity differences can be attributed to changes in the physicochemical properties of IBD‐induced intestinal mucus, including inflammation‐mediated mucus dilution, loosened glycoprotein network structures, and ionic strength changes, which collectively reduce mucus viscous resistance and promote nanocarrier diffusion. This pathological state “advantageous penetration” phenomenon provides unique targeting windows, enabling CAMMR to achieve higher local concentrations at disease sites, thereby improving therapeutic effects while reducing systemic side effects.

The intestinal mucosal adhesion capacity of the delivery system was subsequently studied using elution methods (Figure 4h). Under SIF washing, CAMMR adhesion rates on IBD intestines at 1–5 min were 93.0%, 90.0%, 90.0%, 84.0%, and 87.0%, respectively, demonstrating sustained high adhesion levels, as evidenced by strong fluorescence intensity detected on the mucosa (Figure 4i; Figures S17). Swiss roll fluorescence imaging further confirmed enhanced mucosal adhesion in IBD conditions compared to normal and blank controls, with quantitative analysis showing significantly higher fluorescence intensity in IBD tissues (*p* < 0.0001), indicating preferential binding of CAMMR to inflamed intestinal mucosa (Figure S18).

### 2.5 Biocompatibility and Biodistribution of CAMM

IVIS Lumina III in vivo imaging system tracking revealed distinct time‐dependent distribution patterns of CAMMR throughout the gastrointestinal tract. Fluorescence imaging demonstrated that CAMMR initially accumulated in small intestinal segments of healthy mice within an hour postoral administration, subsequently migrating to the colon over 2–6 h, with near‐complete clearance achieved within 24 h (Figure 4j). Notably, in IBD model mice, compromised intestinal barrier function and increased permeability resulted in preferential accumulation and prolonged retention of CAMMR at colonic inflammatory sites. Particularly in inflamed colon tissues, CAMMR fluorescence signals persisted beyond 24 h, establishing this pathological targeting characteristic as the foundation for precise local drug release. Furthermore, CAMMR distribution remained predominantly confined to the gastrointestinal tract, with minimal detection of systemic circulation or nonspecific accumulation in vital organs. This distribution profile effectively mitigates potential systemic toxicity associated with nanomedicines, demonstrating the distinct advantages of oral targeted delivery systems.

Biocompatibility assessment revealed that Azo‐MOF and CAMM exhibited excellent hemocompatibility across the 0–800 µg mL^−1^ concentration range, with hemolysis rates below 5.0%, meeting established safety standards for biomedical materials (Figures S19 and S20). Additionally, cytotoxicity evaluation demonstrated that Azo‐MOF, CAM (β‐CD‐capped Azo‐MOF without drug loading), and CAMM maintained cell viability above 80.0% across all tested concentrations (5–80 µg mL^−1^), indicating minimal cytotoxic effects. In contrast, free 6‐MP exhibited significant dose‐dependent cytotoxicity, with cell viability decreasing to approximately 20.0% at 80 µg mL^−1^, highlighting the improved biocompatibility of the delivery system (Figure S21). Long‐term toxicity studies following 14 days of continuous oral administration of CAMM showed no mortality or significant body weight loss in experimental animals. Hematological and biochemical parameters remained within normal reference ranges, and histopathological examination of major organs revealed no abnormal changes or injury indicators, further confirming the biocompatibility of this delivery system (Figures S22–S26).

Taken together, CAMM demonstrates favorable biodistribution profiles and safety characteristics. Its selective accumulation at inflammatory sites, combined with excellent biocompatibility, establishes new therapeutic possibilities for targeted IBD treatment.

### 2.6 Synergistic Therapeutic Efficacy in Colitis Models

**FIGURE 5:**
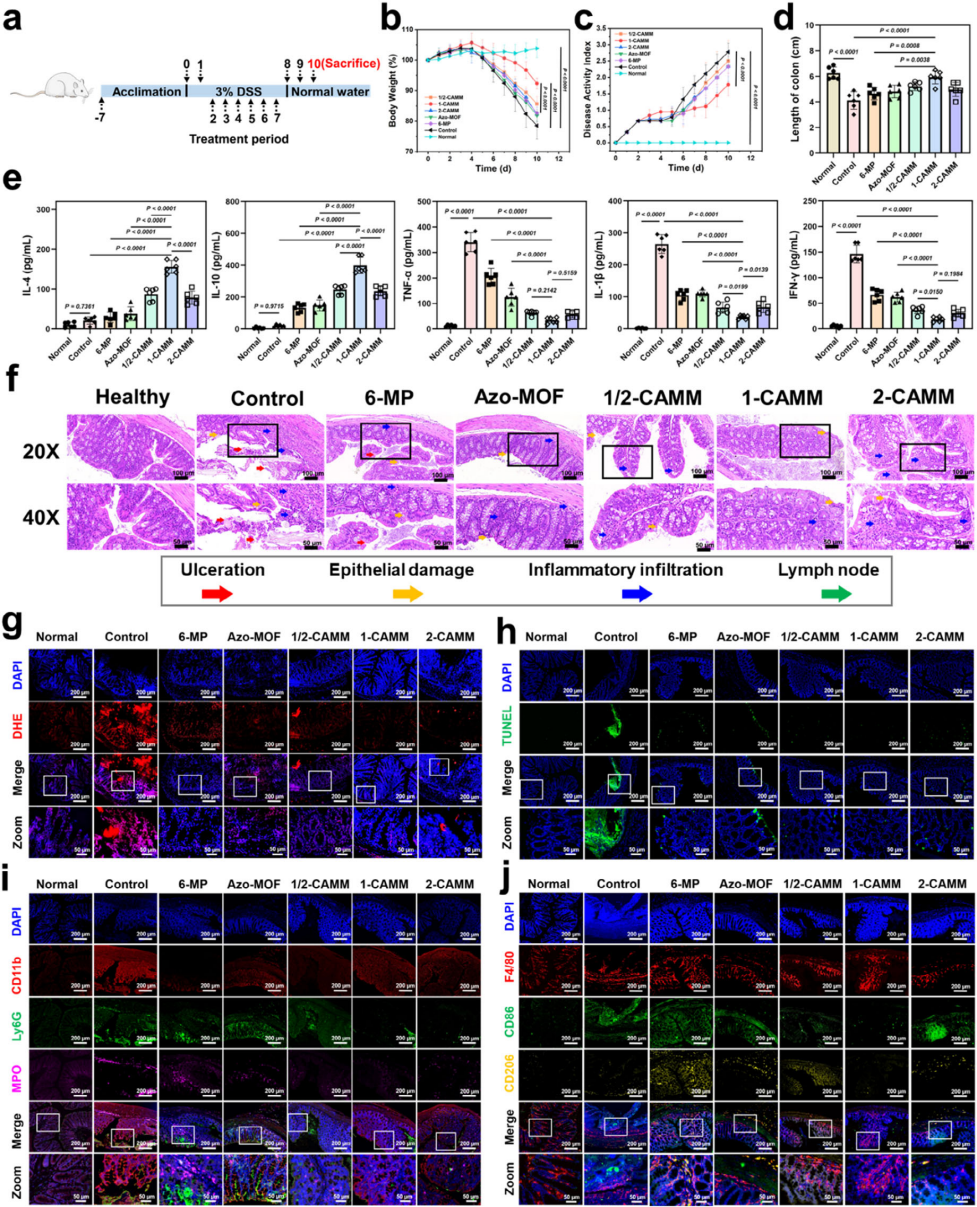
Therapeutic efficacy of CAMM on DSS‐model mice. (a) Schematic illustration of the experimental timeline for the DSS‐induced colitis model and treatment protocol. DSS‐model mice were orally administered with saline (IBD control), 6‐MP, Azo‐MOF, 1/2‐CAMM, 1‐CAMM, and 2‐CAMM, respectively. (b) Body weight changes and (c) Disease activity index (DAI) scores over time. (d) Colon length measurements. (e) Inflammatory cytokine levels in colon tissues. *n* = 6 biologically independent animals. (f) Representative HE staining of colon tissues. (g) DHE staining (blue: nucleus stained with 4′,6‐diamidino‐2‐phenylindole (DAPI), red: ROS). (h) Terminal deoxynucleotidyl transferase dUTP nick end labeling (TUNEL) staining (blue: nucleus stained with DAPI, green: apoptotic cells). (i) MPO, Ly6G, and CD11b staining (blue: nucleus stained with DAPI, purple: MPO‐positive cells, green: neutrophils, red: myeloid cells) for neutrophil infiltration analysis. (j) F4/80, CD86 and CD206 staining (blue: nucleus stained with DAPI, red: macrophages, green: M1 macrophages, yellow: M2 macrophages) for macrophage polarization analysis of colon tissues from DSS‐model mice treated with nanomedicines. *n* = 3 biologically independent animals. Statistical significance indicated by *p* values by a two‐tailed Student's *t*‐test. Data are presented as means ± SD. Source data are provided in the Source Data file.

Based on the CAMM system efficacy in the amelioration of intracellular oxidative stress, a therapeutic evaluation of lesion targeting and controlled drug release was conducted in DSS‐induced mice. Mice received 3.0% DSS in drinking water for 7 days to establish acute IBD models. Therapeutic intervention commenced on day 2 of DSS treatment via daily oral gavage administration until the experimental endpoint (Figure 5a). Experimental groups included saline‐treated IBD controls, healthy mouse controls, and monotherapy groups (6‐MP and Azo‐MOF carrier alone). Additionally, three CAMM treatment groups with different dosages were established (1/2‐CAMM, 1‐CAMM, 2‐CAMM), with the 1‐CAMM dosage determined based on clinical 6‐MP dosing using allometric scaling conversion for mouse body weight, establishing dose‐response relationships and optimal dosing references for clinical translation.

Healthy control mice maintained stable body weight, whereas DSS‐induced mice exhibited significant weight loss accompanied by diarrhea and bloody stools (Figure 5b, c). Following CAMM nanomedicine treatment, all treatment groups showed varying degrees of clinical symptom improvement, with 1/2‐CAMM, 1‐CAMM, and 2‐CAMM recovering to 85.6%, 92.3%, and 82.7% of their initial body weight, respectively. The 1‐CAMM demonstrated optimal therapeutic effects with advantages over 6‐MP monotherapy. Disease activity index (DAI) assessment further confirmed these findings. IBD model mice showed DAI scores of 2.8, which decreased to 2.5, 1.7, and 2.3 following 1/2‐CAMM, 1‐CAMM, and 2‐CAMM treatment, respectively, with 1‐CAMM maintaining the best symptom relief (Figure 5c; Table S2).

On experimental day 10, blood and colonic tissue samples were collected for quantification of inflammatory cytokines and for hematoxylin and eosin (HE), immunofluorescence (IF), and immunohistochemistry (IHC) analyses. Colon length served as an intuitive indicator of colitis severity, with DSS stimulation causing colon shortening (Figure 5d; Figure S27). Mean colon lengths for healthy, IBD control, 6‐MP, Azo‐MOF, 1/2‐CAMM, 1‐CAMM, and 2‐CAMM were 6.2, 4.1, 4.4, 4.9, 5.2, 5.9, and 4.0 cm, respectively, indicating CAMM treatment effectively alleviated colonic tissue damage.

Enzyme‐linked immunosorbent assay (ELISA) quantification of colonic tissue inflammatory cytokines revealed that CAMM treatment restored intestinal immune homeostasis. Pro‐inflammatory cytokines, including tumor necrosis factor‐α (TNF‐α), interleukin‐1β (IL‐1β), interleukin‐6 (IL‐6), interleukin‐17 (IL‐17), monocyte chemoattractant protein‐1 (MCP‐1), and interferon‐γ (IFN‐γ), were reduced, while anti‐inflammatory cytokines interleukin‐4 (IL‐4) and interleukin‐10 (IL‐10) were upregulated (Figure 5e; Figure S28). Intergroup comparative analysis revealed dose‐response characteristics of CAMM nanomedicine. Compared to 6‐MP monotherapy, 1‐CAMM showed advantages in suppressing pro‐inflammatory factor expression and promoting anti‐inflammatory factor secretion, confirming therapeutic enhancement of the nano‐drug delivery system. Both 1/2‐CAMM and 2‐CAMM showed inferior therapeutic effects compared to 1‐CAMM, indicating an optimal therapeutic dose window. Low dosage (1/2‐CAMM) may fail to reach effective drug concentrations for sufficient anti‐inflammatory action, while high dosage (2‐CAMM) may trigger dose‐related adverse reactions or metabolic saturation, diminishing therapeutic effects. To further elucidate the mechanistic basis of the observed anti‐inflammatory effects, the carrier‐mediated activity was validated at the cellular level using LPS‐stimulated RAW 264.7 macrophages. CAM reduced secretion of pro‐inflammatory cytokines TNF‐α, IL‐6, and IL‐1β while promoting anti‐inflammatory cytokine IL‐10 release compared to LPS‐only treatment, demonstrating the intrinsic anti‐inflammatory capacity of Azo‐MOF through ROS scavenging. CAMM achieved superior cytokine suppression, confirming synergistic effects between carrier antioxidant activity and 6‐MP immunomodulation (Figure S29).

HE staining of colonic tissues showed that DSS model mice exhibited typical IBD pathological features, including mucosal surface erosion, epithelial cell necrosis, crypt structure destruction, and massive inflammatory cell infiltration (Figure 5f). Free 6‐MP showed only mild histological improvement, with partial recovery of crypt structure, but persistent severe inflammatory cell infiltration. The Azo‐MOF carrier alone demonstrated protective effects with reduced inflammation but apparent mucosal damage. 1/2‐CAMM and 2‐CAMM showed moderate therapeutic effects, whereas 1‐CAMM demonstrated optimal tissue protection with near‐normal intestinal mucosal structure, regular crypt arrangement, and minimal inflammatory cell infiltration. Histological scoring confirmed this trend with DSS (3.7), 6‐MP (2.7), Azo‐MOF (2.3), 1/2‐CAMM (2.3), 2‐CAMM (1.7), and 1‐CAMM (1.3) (Figure S30; Table S3).

Dihydroethidium (DHE) staining assessment of oxidative stress states revealed differences in ROS scavenging capacity among treatment groups (Figure 5g). Azo‐MOF carrier alone already demonstrated obvious antioxidant effects, with substantially reduced fluorescence intensity compared to the DSS group, confirming the inherent ROS‐scavenging capacity of the nanocarrier. Free 6‐MP showed limited antioxidant effects with strong red fluorescence signals persisting. Among combination preparations, 1/2‐CAMM and 2‐CAMM showed stronger antioxidant effects than the individual components, while 1‐CAMM fluorescence signal intensity decreased to near‐normal control levels, indicating optimal antioxidant protective capacity. TUNEL staining results showed DSS‐induced intestinal epithelial cell apoptosis was reduced in 1‐CAMM (Figure 5h). As key molecular markers of intestinal barrier function, tight junction proteins zonula occludens‐1 (ZO‐1) and claudin expression were downregulated in colitis mice, while 1‐CAMM treatment effectively restored normal expression levels of these proteins, indicating intestinal barrier repair (Figures S31 and S32).

Triple immunofluorescence staining for myeloperoxidase (MPO), lymphocyte antigen 6 complex locus G (Ly6G), and CD11b (integrin αM) enabled precise identification and quantitative analysis of neutrophil infiltration (Figure 5i; Figure S31). MPO serves as a specific enzymatic marker of neutrophils, Ly6G as a characteristic surface glycoprotein, and CD11b as a myeloid cell adhesion molecule. Their co‐localization accurately identifies activated neutrophils. Results showed abundant MPO^+^/Ly6G^+^/CD11b^+^ triple‐positive neutrophils in DSS control colonic tissues, indicating severe acute inflammatory responses. 1‐CAMM treatment reduced neutrophil recruitment and infiltration, with inflammatory cell numbers approaching normal levels, confirming potent anti‐inflammatory effects. F4/80, CD86, and CD206 triple immunofluorescence staining was used to analyze macrophage activation states and polarization phenotypes (Figure 5j; Figure S31). F4/80 serves as a pan‐macrophage marker, CD86 as a M1‐type (classically activated) macrophage characteristic surface molecule, and CD206 as a M2‐type (alternatively activated) macrophage typical marker. CD86 and CD206 expression patterns provide insights into macrophage roles in regulating host inflammatory responses and immune homeostasis. Colitis controls primarily showed F4/80^+^/CD86^+^ M1‐type pro‐inflammatory macrophage accumulation with relatively fewer F4/80^+^/CD206^+^ M2‐type anti‐inflammatory macrophages. Following 1‐CAMM treatment, macrophage phenotypes underwent conversion with reduced M1‐type macrophages and increased M2‐type macrophage proportions, indicating that CAMM effectively promoted macrophage transition from pro‐inflammatory to anti‐inflammatory repair phenotypes, thereby restoring intestinal immune balance.

TNF‐α/IL‐1β dual immunofluorescence staining was used to analyze pro‐inflammatory factor expression across treatment groups. Results showed widespread distribution and strong co‐expression of both pro‐inflammatory factors in colonic tissues from DSS‐model mice, primarily localized to inflammatory cell infiltration areas and damaged mucosal epithelium. Treatment groups showed differences in the suppression of pro‐inflammatory factor expression, with free 6‐MP showing limited inhibitory effects and TNF‐α and IL‐1β expression remaining above normal levels (Figures S33 and S34). 1‐CAMM demonstrated optimal anti‐inflammatory effects, reducing both pro‐inflammatory factors to near‐normal control levels, with only weak signals detected in a few cells at the crypt bases. High‐magnification observations revealed that TNF‐α and IL‐1β were mainly expressed in infiltrating macrophages, neutrophils, and activated intestinal epithelial cells in DSS‐model mice. Following 1‐CAMM treatment, pro‐inflammatory factor‐positive cell numbers decreased, with nearly absent intestinal epithelial cell expression, consistent with our macrophage M1‐to‐M2 polarization conversion results. Compared with the limited therapeutic effects of free 6‐MP, the CAMM delivery system demonstrated superior therapeutic efficacy by integrating snap‐top drug release at inflammatory sites with the intrinsic antioxidant capacity of the nanocarrier, resulting in comprehensive improvements in tissue repair, oxidative stress mitigation, and immune regulation.

### 2.7 Microbiome Modulation and Gut Homeostasis Restoration

**FIGURE 6:**
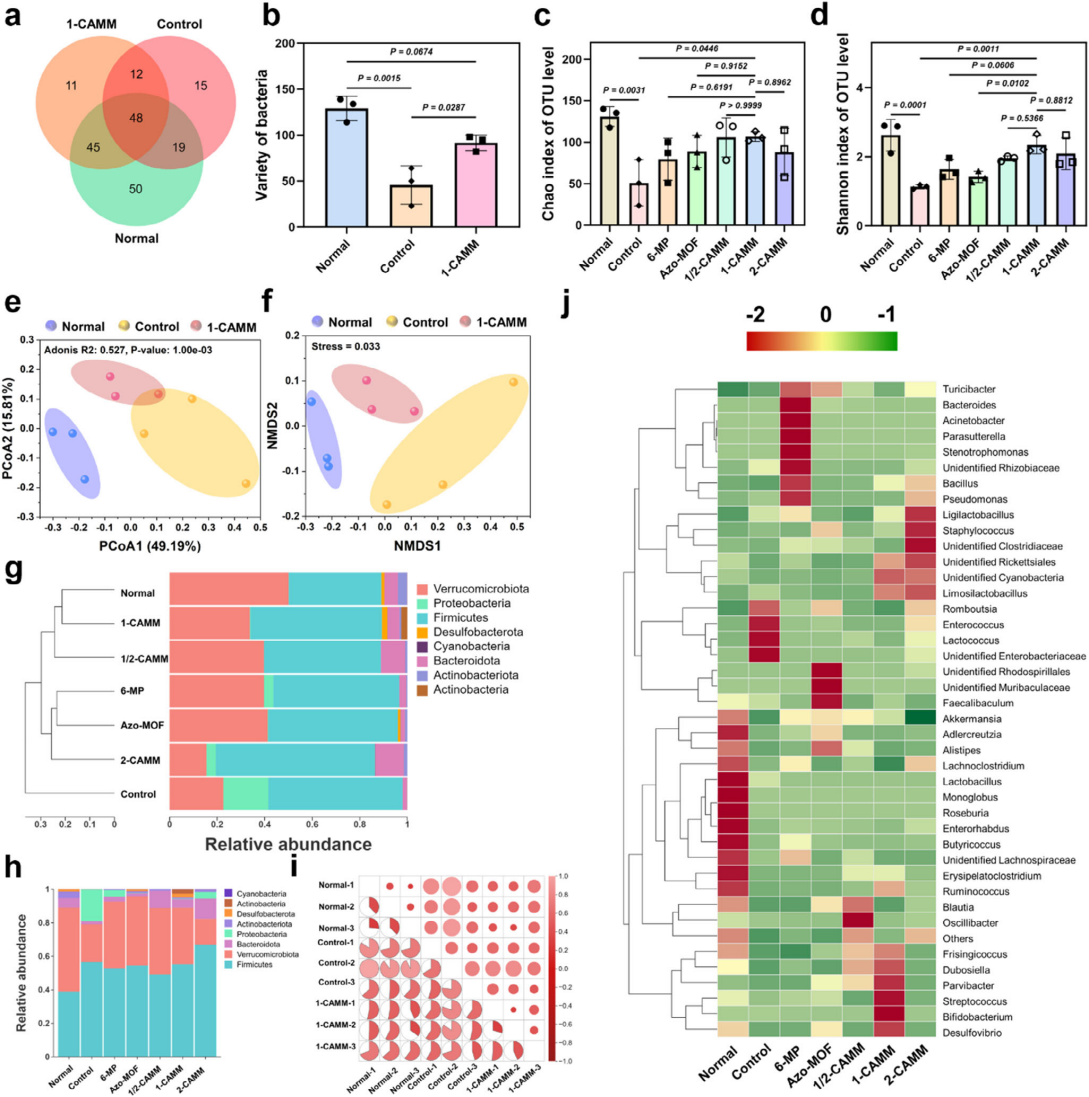
Gut microbiota analysis of CAMM‐treated mice. (a) Venn diagram showing shared and unique bacterial species among Normal, Control, and 1‐CAMM. (b) Total number of bacterial colonies. (c,d) Alpha diversity analysis showing Chao1 and Shannon indices. (e,f) Beta diversity analysis by principal coordinate analysis (PCoA) and nonmetric multidimensional scaling (NMDS) ordination. g) Relative abundance of bacterial phyla across treatment groups. (h) Phylogenetic clustering analysis of gut microbiota composition. (i) Inter‐sample distance analysis showing microbiota similarity patterns. (j) Heatmap of bacterial genera abundance showing taxonomic distribution and clustering relationships. *n* = 3 biologically independent animals. Data are presented as means ± SD. Statistical significance indicated by *p* values by two‐tailed Student's *t*‐test. Source data are provided in the Source Data file.

Accumulating evidence demonstrates intimate associations between intestinal microbial communities and host health, where gut microbiota dysbiosis not only participates in disease pathogenesis but may also influence therapeutic drug efficacy [46]. Given previous experimental confirmation of CAMM therapeutic effects, we conducted 16S rRNA gene sequencing analysis across treatment groups to comprehensively assess CAMM regulatory effects on intestinal microbial community structure and function, particularly exploring microecological contributions to underlying mechanisms. Venn diagram analysis revealed 48 shared operational taxonomic units (OTUs) among the 1‐CAMM, normal, and control groups (Figure 6a,b), indicating that CAMM treatment partially restored normal microbial community structure. Chao1 and Shannon index analyses further quantified these changes (Figure 6c,d), with both indices in 1‐CAMM higher than controls and gradually approaching normal levels. Beta diversity analysis using principal coordinate analysis (PCoA) and non‐metric multidimensional scaling (NMDS) ordination revealed that 1‐CAMM samples were distinct from controls in ordination space, showing clustering trends toward normal controls (Figure 6e,f) and confirming positive regulatory effects at the community level.

Phylum‐level taxonomic analysis revealed specific mechanisms of CAMM intestinal microbiota regulation (Figure 6g,h). Phylogenetic tree analysis showed that 1‐CAMM exhibited relatively close clustering relationships with normal controls, while microbial composition analysis revealed markedly elevated *Proteobacteria* relative abundance in controls, representing typical features of intestinal microbiota dysbiosis. The 1‐CAMM not only alleviated these dysbiotic patterns with partial recovery of *Firmicutes* but also demonstrated a unique significant enrichment of the *Actinobacteria* phylum exceeding normal levels. Inter‐sample distance analysis further validated these findings (Figure 6i), with 1‐CAMM samples showing good consistency and high similarity to normal controls, while control samples exhibited greater variation and increased distances from normal controls.

Genus‐level analysis showed positive effects of 1‐CAMM in enriching beneficial bacterial genera, including *Lactobacillus* and *Bifidobacterium*, while suppressing excessive proliferation of opportunistic pathogenic genera (Figure 6j). Specifically, inflammation‐associated opportunistic pathogens, such as *Enterococcus*, *Staphylococcus*, and certain *Enterobacteriaceae* members, were reduced, with this regulatory pattern most pronounced in 1‐CAMM. These results collectively indicate that CAMM systematically reshapes intestinal microbial communities, particularly by optimizing the relative abundances of key bacterial taxa, thereby providing necessary microecological mechanistic support for therapeutic effects.

## 3 Discussion

Oral therapy for IBD faces a fundamental challenge in balancing safe gastrointestinal transit with efficient site‐specific drug release [47, 48]. Supramolecular gating systems address this by installing stimuli‐responsive molecular valves on nanocarrier surfaces. The conventional design grafts recognition groups as molecular handles onto carriers, then uses macrocycles such as cyclodextrins, calixarenes, or pillarenes as molecular caps to seal pores via host‐guest or electrostatic interactions. Specific stimuli trigger cap dissociation, thereby facilitating drug release. This postmodification strategy has three critical flaws. First, surface functionalization cannot precisely control handle grafting density, orientation, or uniformity, creating heterogeneous gating across particles and yielding batch‐dependent, unpredictable release kinetics. Second, handle‐framework linkages via silanization, amidation, or electrostatic adsorption are vulnerable to gastrointestinal hydrolysis, enzymatic cleavage, and mechanical stress, leading to premature leakage. Third, multistep modifications complicate synthesis and frequently block pores or distort framework structure, diminishing loading capacity and stability. More critically, existing supramolecular gates treat carriers as inert scaffolds focused solely on delivery, ignoring the multifactorial IBD microenvironment. Oxidative stress, microbial dysbiosis, and immune dysregulation form a self‐perpetuating pathological cycle that drug delivery cannot disrupt independently. Integrated platforms combining delivery with microenvironment regulation are essential.

Through rational molecular design, we constructed an integrated intelligent delivery system that overcomes limitations of conventional supramolecular gating. We incorporated azobenzene units directly into the MOF ligand backbone rather than via postmodification. This ligand‐embedded functionalization ensures periodic distribution and chemical equivalence of gating sites throughout the lattice, providing identical β‐CD binding capacity at each pore entrance. Azobenzene anchors to the framework via metal coordination bonds, thereby eliminating the detachment of functional groups common in surface modification. This design maintains drug leakage below 5.0% in SGF at pH 2.0 while achieving over 90.0% cumulative release under azoreductase and alkaline pH at inflamed sites. Azoreductase‐mediated azobond cleavage triggers rapid dissociation of β‐CD, followed by sustained drug release from MOF framework hydrolysis under alkaline conditions. This biphasic release maintains therapeutic drug concentration at inflamed sites beyond 96 h. Azo‐MOF exhibits substantial ROS scavenging capacity, effectively alleviating oxidative stress at inflamed sites. DHE staining shows intense ROS fluorescence in DSS‐induced colonic tissue, while Azo‐MOF and CAMM treatment reduce signal intensity to near‐normal levels, confirming carrier antioxidant activity. This ROS scavenging complements 6‐MP immunosuppression. 6‐MP blocks lymphocyte proliferation by inhibiting purine synthesis, controlling adaptive immune overactivation, while Azo‐MOF neutralizes hydroxyl radicals and superoxide anions to reduce oxidative damage to intestinal epithelial cells. ROS reduction protects membrane lipids and proteins from oxidative modification. It may indirectly suppress proinflammatory cytokine production by decreasing oxidative stress signaling, consistent with the observed downregulation of TNF‐α and IL‐1β. Immunofluorescence staining reveals that CAMM treatment promotes macrophage polarization from the proinflammatory M1 to the anti‐inflammatory M2 phenotype. This shift may partially result from improved redox microenvironment, as M1 macrophage polarization and maintenance depend on elevated intracellular ROS levels.

Structural changes in the gut microbiota following CAMM treatment reflect the combined effects of drug release, relief of oxidative stress, and control of inflammation. 16S rRNA sequencing shows abnormally elevated *Proteobacteria* abundance in DSS model mice, while CAMM treatment significantly reduces *Proteobacteria* and enriches *Lactobacillus* in *Firmicutes* and *Bifidobacterium* in *Actinobacteria*. This microbiota restructuring has therapeutic significance. *Proteobacteria* contain numerous opportunistic pathogens, including Enterococcus and *Enterobacteriaceae*, which are facultative anaerobes capable of utilizing elevated oxygen and nitrate in the inflamed intestinal lumen for respiratory metabolism, gaining growth advantages in inflammatory microenvironments. CAMM restores physiological hypoxic conditions by suppressing inflammation, eliminating the metabolic advantages of these pathogens. Inflammation resolution promotes mucus layer repair, creating colonization niches for obligate anaerobes such as *Lactobacillus* and *Bifidobacterium*. Enrichment of these beneficial bacteria further consolidates therapeutic effects by competitive exclusion of pathogens and reinforcement of the epithelial barrier. This multitarget intervention strategy is essential for chronic diseases like IBD involving complex pathological networks, as single‐target blockade often shows limited efficacy due to compensatory mechanisms or pathway bypass. The functional integration design of CAMM provides a paradigm for developing next‐generation intelligent delivery systems, with potential applications in other gastrointestinal diseases that require simultaneous regulation of inflammation, oxidative stress, and microbial dysbiosis, including Crohn's disease, nonalcoholic steatohepatitis, and inflammation‐associated tumors.

## 4 Conclusions

This study developed a snap‐top azobenzene MOF‐based drug delivery nanosystem that addresses critical challenges in oral IBD therapy through synergistic therapeutic mechanisms. The dual‐responsive mechanism exploiting hypoxic inflammatory environments and alkaline intestinal conditions enables targeted drug release at disease sites, while the ROS‐scavenging capability of Azo‐MOF transforms the carrier from a passive delivery vehicle into an active therapeutic agent. This represents the first application of supramolecular gating systems for IBD treatment, offering an integrated approach combining targeted drug delivery with microenvironmental regulation. This work provides a platform with potential for clinical translation and adaptation to other inflammatory conditions. Future investigations should focus on clinical safety evaluation, scalable production, and exploration of other protein‐based therapeutics to fully realize the potential of this delivery platform for precision medicine.

## 5 Experimental Section

### 5.1 Materials and Methods

#### 5.1.1 Ethical Statement

All procedures involving animals in this study were conducted in accordance with ethical guidelines and received approval from the Animal Ethics Committee of China Medical University under Protocol Number CMU20241548. The experimental mice were housed under specific pathogen‐free conditions as stipulated by the institutional animal care standards.

#### 5.1.2 Animals

Male C57BL/6 mice (6–8 weeks, approximately 20 g) and male Sprague‐Dawley rats (6 weeks, approximately 200 g) were utilized in this investigation under standard humane care protocols. Laboratory animals were housed under controlled environmental conditions with a 12‐h light/dark cycle and provided with ad libitum access to food and water. A 1‐week acclimatization period was implemented before experimental procedures. Despite utilizing only male subjects in our experimental design, the findings have broader applicability beyond sex‐specific limitations.

#### 5.1.3 Materials

All chemicals necessary for Azo‐MOF synthesis were sourced from Energy Chemical. Aladdin Biochemical Technology Co., Ltd provided β‐CD and 6‐MP. Male rat liver microsomes were purchased from Meilunbio Tech. Co., Ltd. Reduced nicotinamide adenine dinucleotide phosphate (NADPH) was from Sigma‐Aldrich. Fluorescent dyes FITC and RITC were acquired from Beijing Bailingwei Technology Co., Ltd. (Beijing, China). Pharmaceutical compounds, including Mes, Ber, Dex, Nor, and Tac, were procured from Beijing Huawei Ruike Chemical Technology Co., Ltd. (Beijing, China)

#### 5.1.4 Software Part

The software used for data collection and analysis in this study included Olex2; Bruker DIFFRAC.SUITE; OMNIC 7.3; Zetasizer Software 7.13; UV Probe 2.7; Tecan i‐control 2.0.10.0; Indigo 2.0.5.0; Slideviewer 2.5; Illumina NovaSeq platform software; GraphPad Prism 9.5.0; ImageJ 1.54f; Origin 2019b.

#### 5.1.5 Preparation of Azo‐MOF

Azo‐ligand (8.44 mg, 0.02 mmol) and Zn(NO3)2·6H2O (11.88 mg, 0.04 mmol) were dissolved in a DMF/H_2_O mixed solvent (v = 7:3, 2 mL total). The reaction mixture was sealed in a glass vial (5 mL) and heated at 120°C for 48 h. Upon cooling to room temperature, yellow rod‐like crystals of Azo‐MOF were obtained. The as‐synthesized crystals underwent a solvent‐exchange process, involving alternate immersion in acetone and CH_2_Cl_2_ at room temperature, to remove residual DMF from the pores. Subsequently, the activated material was isolated as a yellow microcrystalline powder (yield: 17 mg, based on Azo‐ligand).

#### 5.1.6 Preparation of CAMM and CAMMR

Activated Azo‐MOF (100 mg) was dispersed in 6‐MP aqueous solution (1 mM) and subjected to heating at 40°C with sonication for 20 min, followed by stirring under dark conditions for 24 h at 600 rpm. The drug‐loaded material was collected by centrifugation, washed three times with distilled water (10 mL each), and then dried to obtain the intermediate product. Subsequently, the drug‐loaded material was mixed with a saturated aqueous solution of β‐CD (20 mL) and stirred for 24 h at 600 rpm. After centrifugation, the product was washed three times with distilled water (10 mL each) and dried to yield CAMM. For fluorescent labeling, CAMM (100 mg) was dispersed in RITC ethanol solution (1 mM) and stirred in the dark for 12 h. The mixture was centrifuged and repeatedly washed with ethanol until the washing solution became colorless, then air‐dried to obtain CAMMR.

The 6‐MP loading capacity was quantified by UV‐Vis spectrophotometry based on the Beer–Lambert law (Figure S35). The concentration of 6‐MP in the supernatant before and after loading was determined using the standard calibration curve (y = 17.26132x + 0.08058, R^2^ = 0.99978), and the drug loading capacity was calculated to be 147 mg g^−1^. The encapsulation efficiency was calculated using the following equation:

(1) $$\mathrm{Encapsulation}\;\mathrm{efficiency}\;\;\left(\%\right)=\frac{M_{0}-M_{S}}{M_{0}}\;\times 100$$

where *M_0_* is the initial amount of 6‐MP added for loading, and *M_S_* is the amount of 6‐MP remaining in the supernatant after loading. The encapsulation efficiency at the optimal loading concentration (1 mM) was determined to be 83.5%.

#### 5.1.7 Drug Release Kinetics

Drug release experiments were conducted using a cuvette‐based spectrophotometric method. CAMM (1 mg) was fixed at one corner of a cuvette and gently pressed with a flat spatula to secure the material. A 3 × 5 mm magnetic stirring bar was positioned at the diagonal corner opposite to the sample (Figure S36). Test solutions (SIF pH 6.8, SGF pH 2.0, SCF pH 7.4, pH 6.8 PBS buffer containing SDT, and pH 6.8 PBS buffer containing rat liver microsomes and NADPH) were slowly added along one edge of the cuvette. The absorbance at 320 nm was measured at predetermined time intervals (0, 1, 2, 4, 6, 8, 10, 12, 24, 36, 48, 60, 72, and 96 h). The relative release percentage of 6‐MP was calculated using the following equation:

(2) $$\mathrm{Relative}\;\mathrm{release}\;\mathrm{percentage}\;\;\left(\%\right)=\frac{M_{t}}{M_{\infty }}\;\times 100=\frac{A_{t}}{A_{\infty }}\;\times 100$$

where the *M_t_* is the amount of cargo (6‐MP) released from the materials at time *t*, *M_∞_* is the amount of cargo (6‐MP) released from the materials at time infinity. According to Beer's Law, the amount of cargo (6‐MP) released from the materials (*M_t_*, *M_∞_*) was directly proportional to the UV–Vis absorbance (*A_t_*, *A_∞_*).

For hypoxia‐responsive drug release evaluation, two test solutions were prepared: (1) pH 6.8 PBS buffer containing SDT (1, 10, and 100 mM) as a chemical mimic of azoreductase, and (2) pH 6.8 PBS buffer containing rat liver microsomes (3.0 mg mL^−1^) and NADPH (40 µM) to simulate the physiological hypoxic reducing environment. In the rat liver microsome system, hypoxic conditions were established by purging the test solution with nitrogen gas for 45 min prior to the addition of CAMM, thereby removing dissolved oxygen and creating a reducing environment. The cuvette was then sealed with a rubber septum to maintain the hypoxic atmosphere throughout the experiment. All microsome‐based release studies were conducted at 37°C to mimic physiological temperature. Control experiments were performed in the absence of microsomes or NADPH to confirm the enzyme‐dependent nature of the hypoxia‐responsive release mechanism. For the SDT system, the test solution was prepared immediately before use by dissolving SDT in degassed pH 6.8 PBS buffer under a nitrogen atmosphere. The experiments were conducted according to the same spectrophotometric protocol described above, with absorbance measurements at 320 nm recorded at the predetermined time intervals.

#### 5.1.8 In Vitro Antioxidant Assay

DPPH Radical Scavenging Assay: The free radical scavenging capacity of Azo‐MOF was evaluated using the DPPH method. Various concentrations of Azo‐MOF (0.1, 0.25, 0.5, 1.0, 1.5 mg mL^−1^, *n *= 3) in ethanol (2 mL) were mixed with DPPH solution (2 mL, 100 µg mL^−1^) and incubated in the dark. Following incubation periods of 2 h, absorbance values were measured at 520 nm using a spectrophotometer.

Superoxide Anion Scavenging Assay: Superoxide anion elimination capacity was assessed using the xanthine/xanthine oxidase system with WST‐1 reagent. The assay utilized the principle that WST‐1 reacts with superoxide anions generated by xanthine oxidase to produce water‐soluble formazan dye, which can be inhibited by antioxidant compounds. Different concentrations of Azo‐MOF in PBS (pH 7.4, 0.1, 0.25, 0.5, 1.0, 1.5 mg mL^−1^, 20 µL, *n* = 3), enzyme working solution (20 µL), and substrate application solution (200 µL) were combined in wells, incubated at 37°C for 20 min, and absorbance was recorded at 450 nm.

Hydroxyl Radical Scavenging Assay: Hydroxyl radical elimination was determined using the Fenton reaction system, where H_2_O_2_/Fe^2+^ generates hydroxyl radicals that are captured by salicylic acid to form 2,3‐dihydroxybenzoic acid. The reaction mixture contained substrate A (100 µL), substrate B (100 µL), distilled water (480 µL), substrate C (200 µL), and varying concentrations of Azo‐MOF (0.1, 0.25, 0.5, 1.0, 1.5 mg mL^−1^, 20 µL, *n *= 3). After mixing and incubation at 37°C for 20 min, the optical density was measured at 510 nm.

#### 5.1.9 EPR Analysis

EPR spectroscopy was employed to assess the radical scavenging capacity of Azo‐MOF using a Bruker EMXPlus spectrometer. For hydroxyl radical detection, the Fenton reaction system was established by combining ferrous sulfate (1.0 mM) with hydrogen peroxide (5 mM) at pH 4.0 to generate •OH. DMPO spin trap, freshly generated hydroxyl radicals, and Azo‐MOF (10 mg mL^−1^, 100 µL) were incubated together at 37°C for 2 min, followed by EPR spectral recording of DMPO/•OH adducts. All measurements were performed in triplicate.

For superoxide anion analysis, the enzymatic reaction system consisting of xanthine (0.5 mM) and xanthine oxidase (0.2 mU) was used to produce •O_2_^−^ radicals. After 2‐min incubation at 37°C with DMPO spin trap and Azo‐MOF (10 mg mL^−1^, 100 µL), EPR spectra of DMPO/•O_2_^−^ complexes were acquired. Each experiment was repeated three times to ensure reproducibility.

#### 5.1.10 Intracellular ROS Detection

Intracellular ROS levels were determined using the DCFH‑DA fluorescent probe. RAW 264.7 cells were seeded in 24‑well plates at a density of 10^5^ cells per well (*n* = 3) and incubated at 37°C under 5% CO_2_ for 12 h. The medium was then replaced with medium containing nanomedicines (25/50/100 µg mL^−1^), and the cells were incubated for a further 4 h, followed by stimulation with LPS at a final concentration of 20 µg mL^−1^ for 4 h to induce oxidative stress. Cells treated with DMEM medium alone served as the negative control. After incubation, the culture medium was removed, and the cells were washed twice with PBS. Subsequently, the cells were stained with 10 µM DCFH‑DA (Dalian Meilun Biotechnology Co., China) for 30 min at 37°C in the dark. Fluorescence images were acquired by confocal laser scanning microscopy (CLSM) to visualize and quantify intracellular ROS levels.

#### 5.1.11 Intestinal Mucus Penetration

To simulate the viscoelastic properties of intestinal mucus, various media were prepared, including hydroxyethyl cellulose (0.5% HEC), glycerol (80.0% Gly), glycerol supplemented with L‐alanine (0.5 mg mL^−1^), glycerol containing hydrogen peroxide (0.1 mM), and freshly extracted rat mucus samples. Each medium (1.5 mL) was loaded into syringes, and SIF suspension containing CAMMR (1 mg mL^−1^, 0.5 mL, *n* = 3) was gently layered on top to allow free diffusion. After 6 h of incubation, images were captured and bottom samples (0.2 mL) were collected following careful washing. Fluorescence intensity measurements (Ex/Em: 550/620 nm) were performed to determine particle penetration ratios using the equation:

(3) $$\mathrm{Particle}\;\mathrm{penetration}\;\mathrm{ratio}\;\;\left(\%\right)=\frac{I_{i}}{\sum_{i=1}^{5}I_{i}}\;\times 100$$

where *I_i_* represents the fluorescence intensity of nanoparticles that penetrated each 2 mL segment from bottom to top.

CLSM Analysis: Fresh colon segments from healthy and DSS‐treated rats (4.0% DSS for 7 days) were gently washed with SIF and sectioned into 4 cm pieces. After ligation at both ends, segments were incubated with FITC solution (10 µg mL^−1^, 500 µL) in Tyrode solution at 37°C for 10 min. Following FITC removal, CAMMR suspension in SIF (100 µg mL^−1^, 100 µL, *n* = 3) was injected and incubated at 37°C for 30 min. Colonic tissues were opened along the midline, washed with SIF, and positioned flat in confocal dishes (luminal side down) for z‐stack imaging using CLSM (THUNDER Image DMi8, Leica, Germany).

#### 5.1.12 Multiple Particle Tracking

Brownian motion trajectories of CAMMR in intestinal mucus were analyzed using multiparticle tracking methodology. Fresh mucus samples (200 µL) collected from the rat and DSS‐model rat intestinal lumens were incubated with CAMMR solution (200 µg mL^−1^, 10 µL, *n* = 3) for 30 min at 37°C. Microscopic samples (20 µL) were placed on dishes for trajectory capture using CLSM at a 37 fps recording rate. Ten‐minute videos were processed with ImageJ software to calculate MSD and D_eff_:

(4) $$SD_{\tau }={\left(x_{t+\tau }-x_{t}\right)}^{2}+{\left(y_{t+\tau }-y_{t}\right)}^{2}$$

(5) $$D_{eff}=\frac{MSD}{4\tau }$$

#### 5.1.13 Intestinal Mucosal Adhesion Assessment

Colon tissues from fasted SD rats and DSS‐model rats were isolated, washed with SIF, and sectioned into 1 × 4 cm segments on slides. CAMMR particles (10 mg, *n* = 3) were uniformly distributed on tissue surfaces and incubated at 37°C for 15 min on a 45° platform. SIF elution was performed for 5 min to remove nonadherent particles, and eluent fluorescence intensity was measured (Ex/Em: 570/595 nm). Postelution mucosal fluorescence was documented using the Living Image System (Night OWL II LB 983, Germany) with semi‐quantitative analysis via indiGo software. Tissues were subsequently fixed in formalin, embedded in OCT, cryosectioned, DAPI‐stained, and examined by CLSM.

#### 5.1.14 Intestinal Uptake and Retention Studies

Healthy and DSS‐model mice received oral administration of CAMMR (100 mg kg^−1^, *n* = 3). After 2 h, colonic tissues were harvested, formalin‐fixed, OCT‐embedded, cryosectioned, DAPI‐stained, and analyzed by CLSM. For gastrointestinal retention assessment, fasted mice were administered CAMMR (100 mg kg^−1^, *n* = 3) and complete GIT, along with major organs, were collected at predetermined intervals for ex vivo fluorescence imaging and semi‐quantitative intensity analysis.

#### 5.1.15 Biocompatibility

Hemolysis Assay: Blood compatibility of Azo‐MOF and CAMM was assessed through hemolysis testing. Following overnight fasting, venous blood samples were collected from SD rats and processed by centrifugation and washing to isolate red blood cells (RBCs). The obtained RBCs were resuspended in sterile saline to prepare 2.0% (v/v) cell suspension. Equal volumes of RBC suspension (2 mL) were combined with varying concentrations of Azo‐MOF and CAMM in normal saline (*n* = 3). Control groups consisted of RBC suspension mixed with equal volumes of normal saline (negative control) and deionized water (positive control). After 4 h of incubation, samples were centrifuged, and the supernatant absorbance was measured using UV–Vis spectrophotometry at 540 nm.

In Vivo Toxicity Evaluation: Systemic toxicity of CAMM was evaluated through acute toxicity studies. Mice received daily oral administration of CAMM at double therapeutic dose (200 mg kg^−1^, *n* = 3) for 7 consecutive days, with body weight monitoring every two days. On day 14, whole blood and serum samples were collected for hematological analysis (BC‐2800vet, Mindray, China) and biochemical evaluation (poch‐100i, Sysmex, Japan), respectively. Major organs, including heart, liver, spleen, lungs, kidneys, and gastrointestinal tract, were harvested, weighed, and processed for histological examination using HE staining. Additionally, intestinal tissues were immunostained with ZO‐1 and occludin antibodies to assess barrier function integrity.

#### 5.1.16 Cytotoxicity Assay

The cytotoxicity of nanomedicines was evaluated using the CCK‐8 assay. RAW 264.7 cells were seeded in 96‐well plates at a density of 1 × 10^4^ cells per well (*n* = 3) and cultured at 37°C with 5% CO_2_ for 12 h to allow adherence. The culture medium was then replaced with fresh medium containing various concentrations of test compounds (Azo‐MOF, 6‐MP, or CAMM at 5, 10, 20, 40, 60, and 80 µg mL^−1^). Control wells received culture medium only. After 24 h incubation, the medium was removed and replaced with CCK‐8 working solution (Glpbio, USA) according to the manufacturer's instructions. The absorbance at 450 nm was measured using a microplate reader, and cell viability was calculated using the following formula:

(6) $$\mathrm{Cell}\;\mathrm{viability}\;\;\left(\%\right)=\frac{A_{e}}{A_{c}}\times 100\%$$

where *A_e_* represents the absorbance of the experimental groups and *A_c_* represents the absorbance of the control group.

#### 5.1.17 In Vitro Anti‐Inflammatory Assay

The anti‐inflammatory effects of nanomedicines were evaluated in LPS‐stimulated RAW 264.7 macrophages. Cells were seeded in T25 culture flasks and cultured at 37°C with 5% CO_2_ for 12 h to allow adherence. Cells were then treated with nanomedicines (6‐MP, Azo‐MOF, CAM, or CAMM at 60 µg mL^−1^, *n* = 3) for 12 h, followed by stimulation with LPS (20 µg mL^−1^ final concentration) for an additional 6 h. Control groups received DMEM medium only, while the LPS group received LPS stimulation without nanomedicine treatment. Cell culture supernatants were collected and centrifuged to remove cellular debris. Levels of pro‐inflammatory cytokines (TNF‐α, IL‐1β, IL‐6) and anti‐inflammatory cytokine (IL‐10) were quantified using commercial ELISA kits (Elabscience, Wuhan) according to the manufacturer's protocols.

#### 5.1.18 DSS‐Induced Acute Colitis Model

Acute ulcerative colitis was induced in C57BL/6 mice by replacing normal drinking water with 3.0% (w/v) DSS solution for 7 consecutive days. Based on clinical 6‐MP dosage (0.84 mg kg^−1^) [49] and allometric scaling calculations for mice (conversion factor: 70 kg × 0.0025/0.02 kg), the equivalent mouse dose was determined as 7.644 mg kg^−1^. Using an average mouse weight of 20 g, the corresponding material doses were calculated for different treatment groups.

Experimental Groups and Dosing: Based on the clinical 6‐MP dosage of 0.84 mg kg^−1^ reported in literature, equivalent mouse doses were calculated using allometric scaling. The treatment groups were designed as follows:

Free 6‐MP group: 7.644 mg kg^−1^ (equivalent to 0.153 mg pure 6‐MP per mouse), representing the clinical therapeutic dose

Azo‐MOF carrier group: 0.888 mg per mouse (equivalent dose of carrier material after subtracting drug loading capacity), providing precise control for carrier effects

1/2‐CAMM group: 3.822 mg kg^−1^ (0.520 mg CAMM per mouse, containing 0.076 mg 6‐MP)
1‐CAMM group: 7.644 mg kg^−1^ (1.041 mg CAMM per mouse, containing 0.153 mg 6‐MP)
2‐CAMM group: 15.288 mg kg^−1^ (2.080 mg CAMM per mouse, containing 0.306 mg 6‐MP)

Given the drug loading capacity of 147 mg g^−1^, the Azo‐MOF carrier control group received the calculated carrier mass (0.888 mg) to ensure accurate assessment of carrier‐specific effects independent of drug activity.

Assessment Parameters: Body weight, water intake, and Disease Activity Index (DAI) scores based on body weight loss, fecal blood, and stool consistency were recorded daily throughout the experimental period. Following a 2‐day recovery phase, mice were euthanized on day 10 for sample collection.

Sample Collection and Analysis: Colon tissues were harvested for length measurement, followed by fixation and embedding for histopathological examination. Tissue sections were processed for HE staining and immunofluorescence analysis using antibodies against tight junction proteins (ZO‐1, Occludin), macrophage markers (CD86, CD206, F4/80), neutrophil markers (MPO, Ly6G, CD11b), and inflammatory cytokines (TNF‐α, IL‐1β) for immunohistochemistry studies.

Colon tissue homogenates were prepared in pH 7.4 PBS (1:9 ratio) for cytokine quantification. Levels of TNF‐α, IL‐1β, IL‐4, IL‐6, IL‐10, IL‐17, MCP‐1, and IFN‐γ were determined using commercial ELISA kits (Elabscience, Wuhan) according to the manufacturer's protocols.

Microbiome Analysis: Fecal samples were collected for 16S rRNA gene sequencing to assess gut microbiota composition and diversity changes following treatment interventions. Samples were processed for high‐throughput sequencing and bioinformatics analysis to evaluate microbial community structure and taxonomic distribution.

#### 5.1.19 Statistical Analysis

Three parallel replicates were used for all in vitro experiments, whereas six parallel groups were employed for animal studies. Results are expressed as mean ± standard deviation (SD). Two‐tailed Student *t*‐test and one‐way ANOVA were applied for statistical comparisons between groups. *p*‐values less than 0.05 were considered statistically significant. Throughout this study, specific *p*‐values are provided in the text and figures instead of symbolic representations to ensure precise statistical reporting. Statistical analyses were performed using GraphPad Prism 9.5.0, with additional data processing conducted using ImageJ 1.54f and Origin 2019b.

[CCDC 2421494 contains the supplementary crystallographic data for this paper. The data can be obtained free of charge from The Cambridge Crystallographic Data Centre via www.ccdc.cam.ac.uk/data_request/cif].

## Conflicts of Interest

The authors declare no conflicts of interest.

## Supporting information




**Supporting File 1**: advs75122‐sup‐0001‐SuppMat.pdf.

**Supporting File 2**: advs75122‐sup‐0002‐VideoS1‐S4.zip.

**Supporting File 3**: advs75122‐sup‐0003‐DataFile.zip.

## Data Availability

The data that support the findings of this study are available in the supplementary material of this article.
