# Supplementary material for: Construction of a MOF‐Based Snap‐Top Delivery Nanosystem for Powerful Dual‐Responsive Synergistic Colitis Treatment
Source: Adv Sci (Weinh). 2026 Apr 2;13(36):e24174. doi: 10.1002/advs.202524174 (PMC13317788; doi:10.1002/advs.202524174)
Supplement: Supplementary file 1 — Supporting File 1: advs75122‐sup‐0001‐SuppMat.pdf. [file ADVS-13-e24174-s002.pdf]

Supporting Information

Construction of a MOF-Based Snap-Top Delivery Nanosystem for Powerful Dual-Responsive Synergistic Colitis Treatment

Xin Li, Miao Xu, Heran Li, Huan Jiang,* Xin Wang, Lianjun Ma, and Ying-Wei Yang**

Methods

Synthesis of Azo-ligand (3):

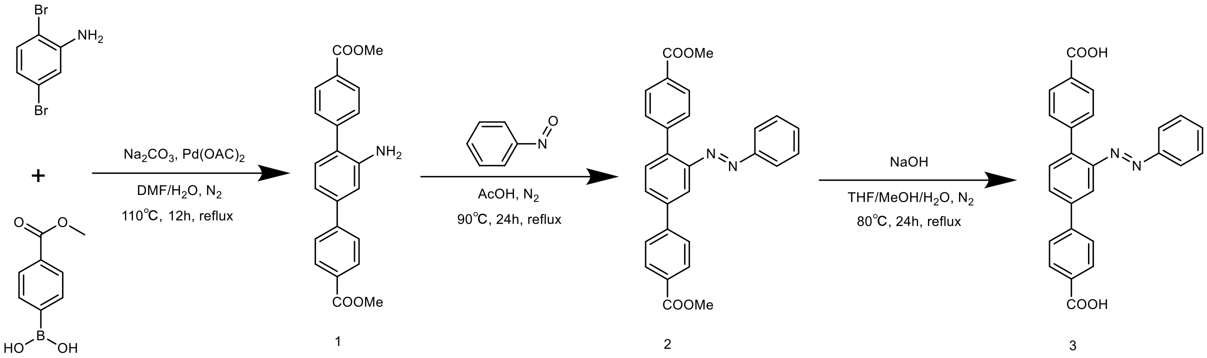

Figure S1. Synthesis route of Azo-ligand (3).

Synthesis of 1. Under a nitrogen atmosphere, 2,5-dibromoaniline (1.26 g, 5.00 mmol), 4-methoxycarbonylphenylboronic acid (5.40 g, 30.00 mmol), palladium acetate (9.60 mg, 43.00 mmol), and sodium carbonate (2.26 g, 21.30 mmol) were added to a deoxygenated solution of N, N-dimethylformamide (16 mL) and water (19 mL) in a 100 mL Schlenk flask. The reaction mixture was stirred at 110 ºC for 12 hours. After cooling to room temperature, an excess of water was added to the solution. The resulting yellow powder (1.56 g, yield: 87%, based on 2,5-dibromoaniline) was collected by filtration, washed with water, and dried under vacuum. ^{1}H NMR (400 MHz, DMSO-d_6, 22°C, ppm): δ = 8.05-8.03 (m, 4H), 7.78 (d, 2H), 7.65 (d, 2H), 7.17 (t, 1H), 7.15 (s, 1H), 7.03 (dd, 1H), 5.12 (s, 2H), 3.88 (d, 6H).

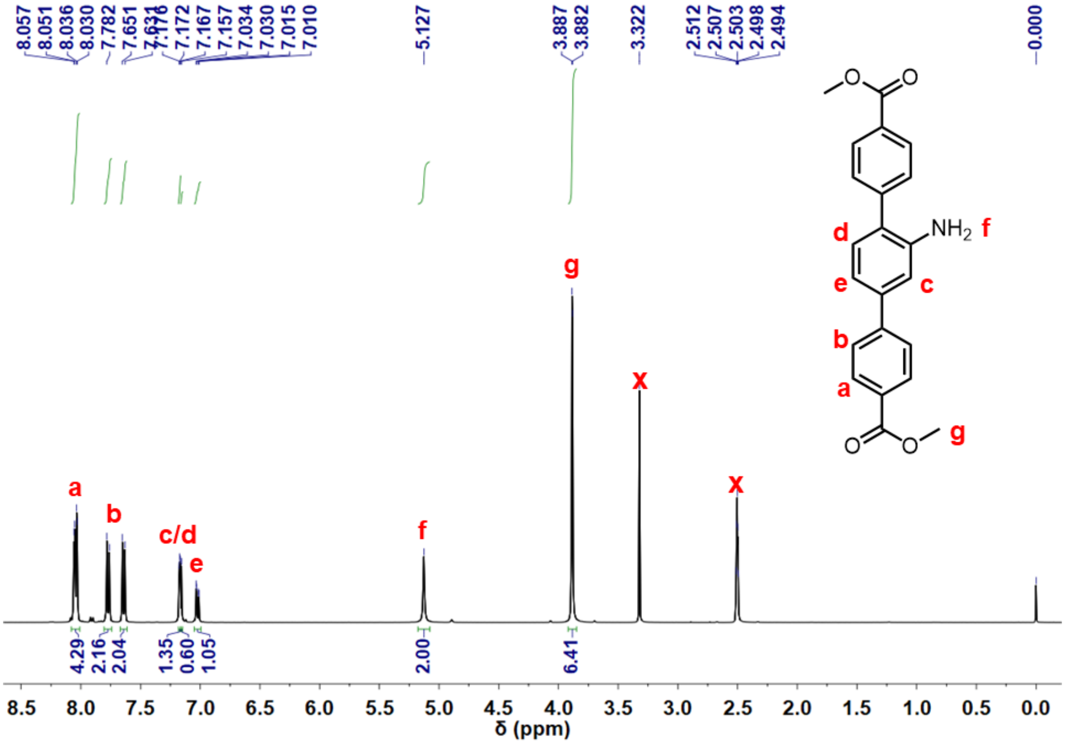

Figure S2. ^{1}H NMR spectrum (DMSO-d_6, 400 MHz, 22 °C) of **1**.

Synthesis of 2. To a suspension of **1** (1.89 g, 5.00 mmol) in AcOH (100 mL), was added nitrosobenzene (2.14 g, 20.00 mmol), and the mixture was stirred at 90°C for 24 h under N_2. The reaction mixture was allowed to cool to room temperature, and then evaporated to dryness under reduced pressure. The residue was suspended in water (50 mL), and an insoluble fraction was separated by filtration and dried under reduced pressure. A light yellow solid substance thus obtained was washed with hot EtOH and subjected to recrystallization from THF, affording a yellow solid substance (yield: 1.99 g, 80% based on **1**). ^{1}H NMR (400 MHz, $CDCl_3$, 22°C, ppm): δ = 8.16-8.11 (m, 4H), 8.05 (d, 1H), 7.83-7.81 (m, 2H), 7.80 (d, 2H), 7.77 (s, 1H), 7.70 (d, 1H), 7.61 (d, 2H), 7.49-7.47 (m, 3H), 3.96 (d, 6H).

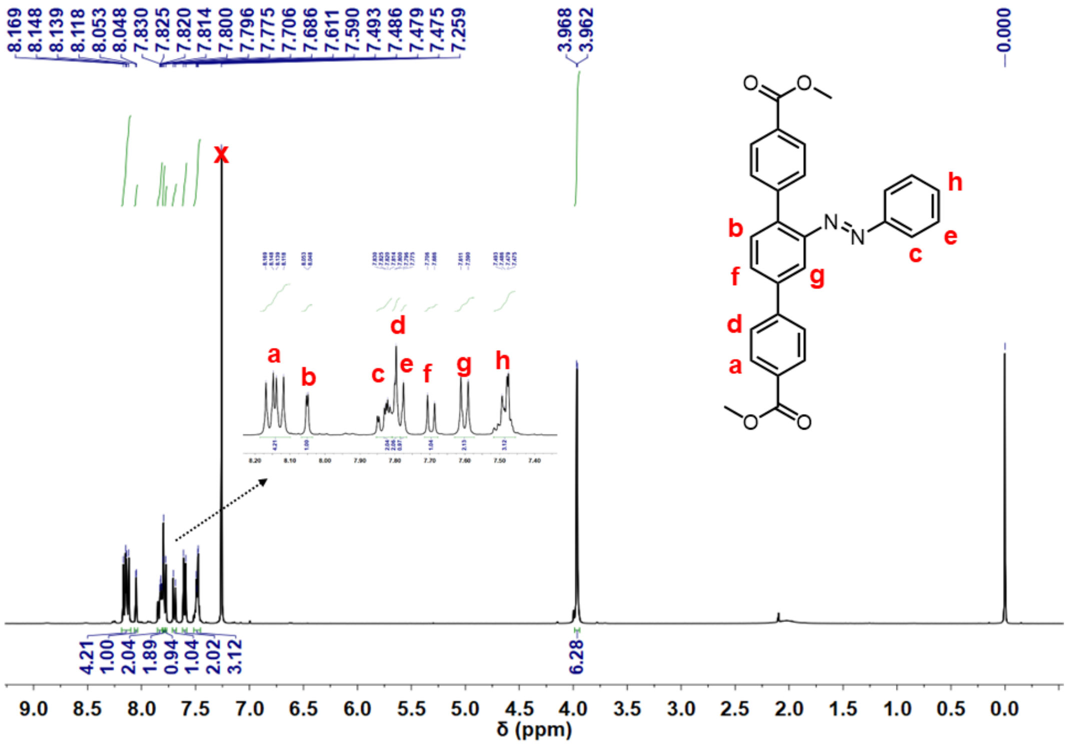

Figure S3. ^{1}H NMR spectrum ($CDCl_3$, 400 MHz, 22 °C) of compound **2**.

2.1.3 Synthesis of Compound 3

To a THF (40 mL)/MeOH (40 mL) solution of **2** (0.99 g, 2.24 mmol) was added an aqueous solution (40 mL) of KOH (4.60 g, 82.00 mmol), and the mixture was refluxed for 24 h. The reaction mixture was allowed to cool to room temperature and evaporated to dryness under reduced pressure. The residue was dissolved in water (100 mL) at 70 °C, and the resulting reddish orange solution was acidified with HCl (1 M) until no further precipitate formed (pH ~ 3). A orange-colored solid substance was collected by filtration, washed with a large volume of water, and dried under reduced pressure (yield: 0.94 g, 99% based on **2**). ^{1}H NMR (400 MHz, DMSO-d_6, 22°C, ppm): δ = 13.04 (br, 2H), 8.10-8.02 (m, 6H), 7.94 (d, 2H), 7.84 (d, 1H), 7.80 (dd, 2H), 7.67 (d, 2H), 7.59-7.56 (m, 3H).

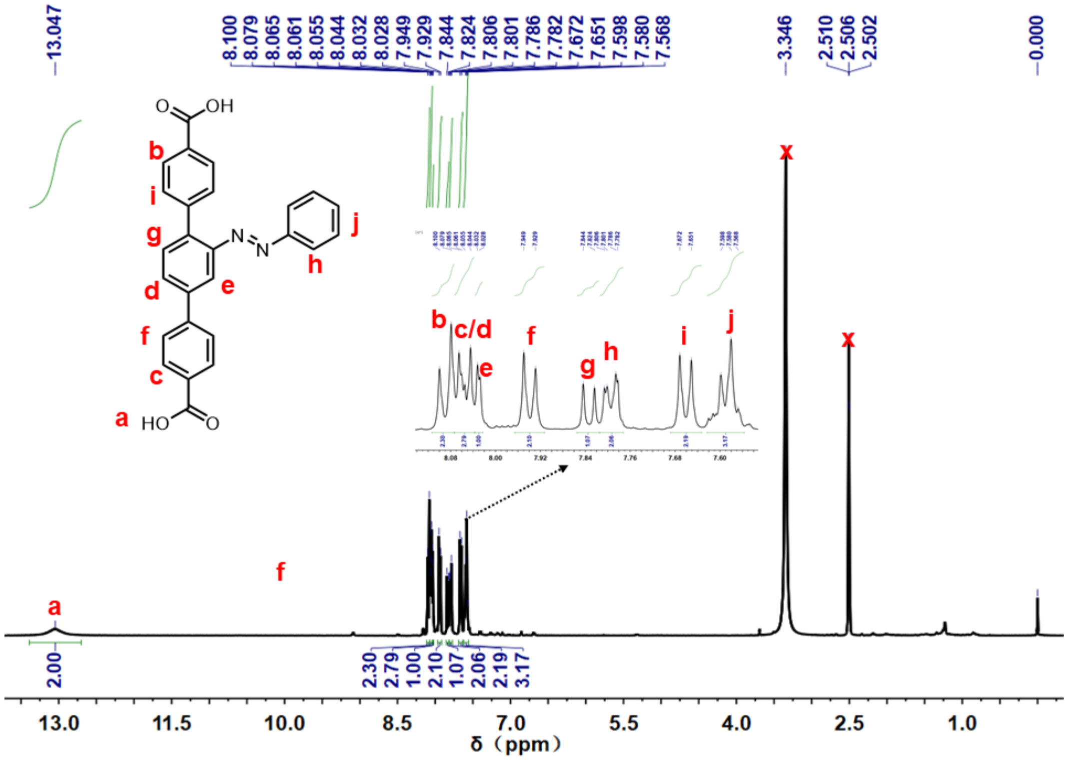

Figure S4. ^{1}H NMR spectrum (DMSO-d_6, 400 MHz, 22 °C) of compound **3**.

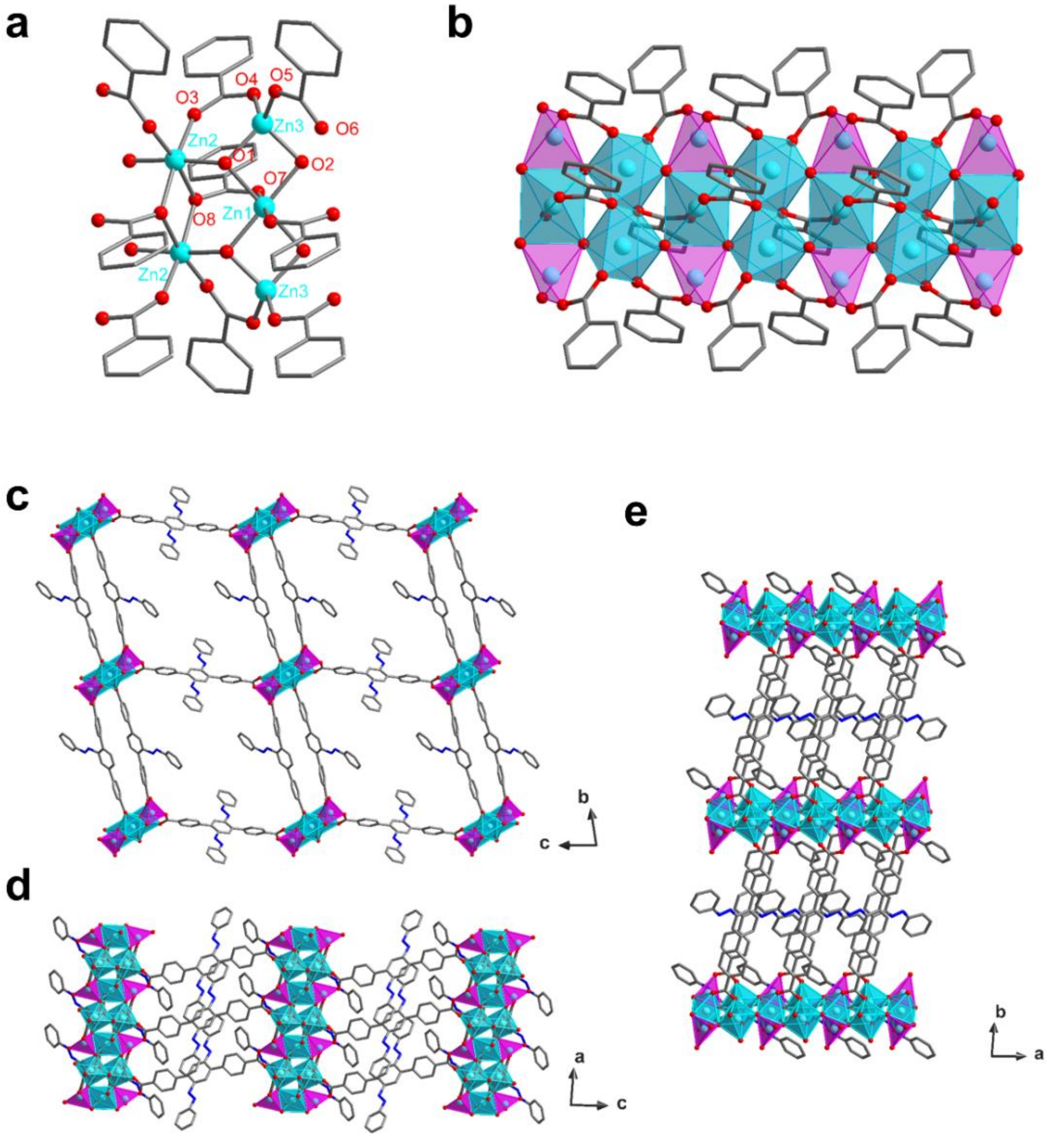

Figure S5. **a,** Coordination environment of zinc metal centers in Azo-MOF. **b,** One-dimensional chain structure formed by trinuclear zinc-oxo clusters extending along the a-axis. **c-e,** 3D framework structure of AZO-MOF viewed along the a-, b- and c-axis, respectively. Purple polyhedra represent [ZnO_4] tetrahedral configurations, while blue polyhedra indicate [ZnO_6] octahedral coordination environments. Hydrogen atoms and portions of the ligand structure have been omitted for clarity.

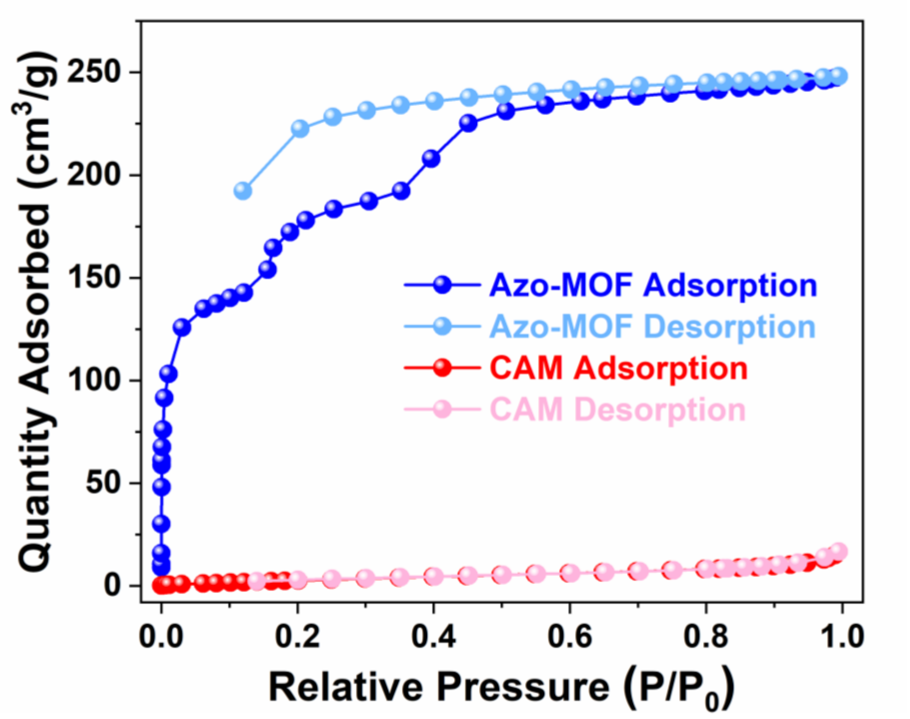

Figure S6. N_2 adsorption-desorption isotherms measured at 77 K for Azo-MOF (blue) and CAM (red, drug-free material with only *β*-CD capping).

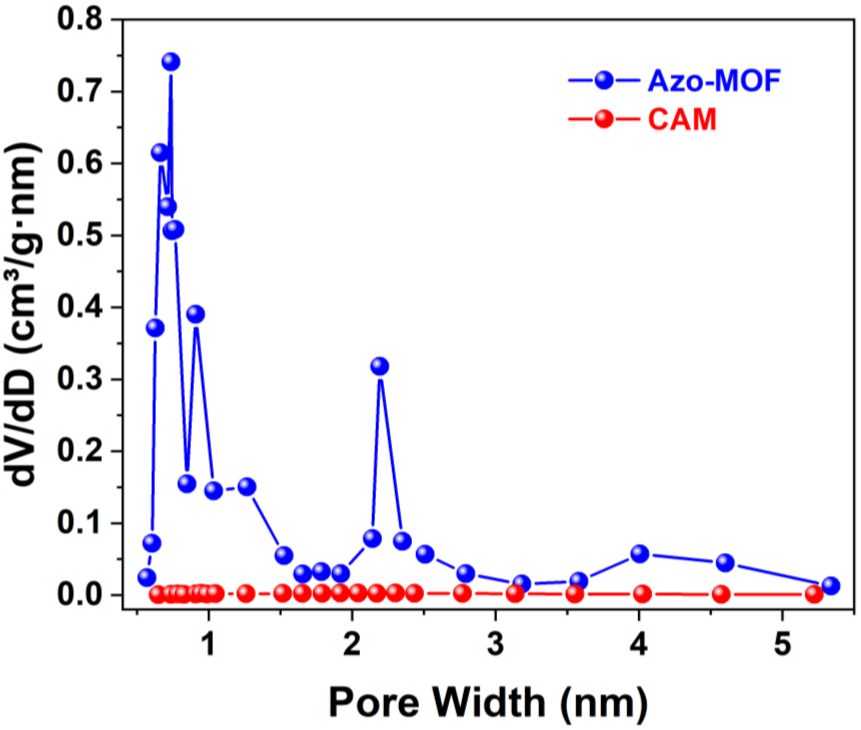

Figure S7. Pore size distribution of Azo-MOF (blue) and CAM (red, drug-free material with only β-CD capping).

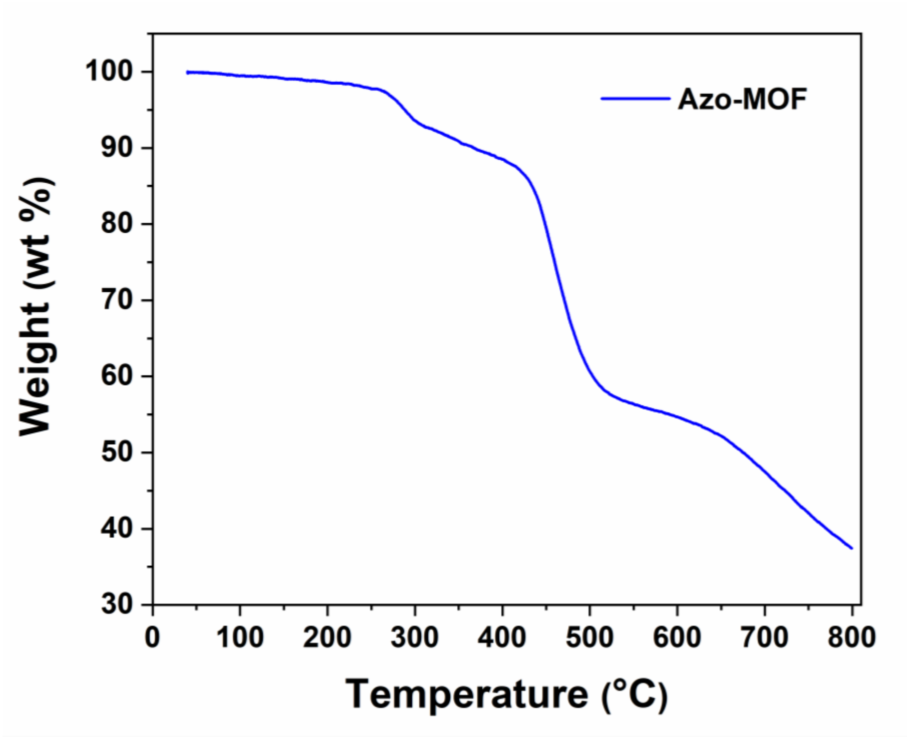

Figure S8. The TGA curve of Azo-MOF.

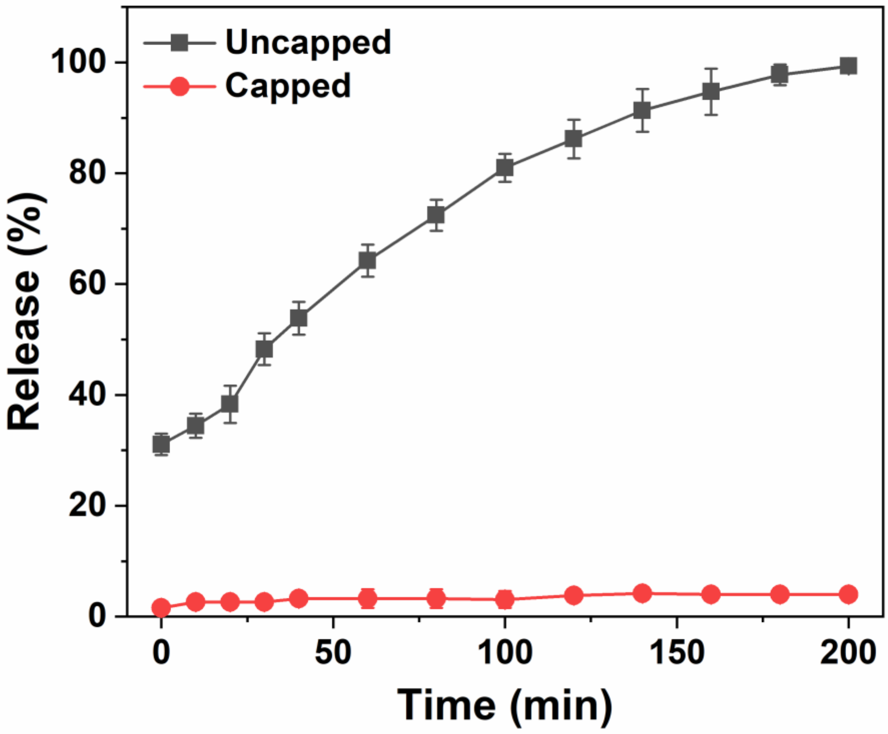

Figure S9. In vitro drug release profiles of uncapped and capped drug delivery systems in SGF (pH 2.0). Data are presented as means ± SD (*n* = 3). Source data are provided in the Source Data file.

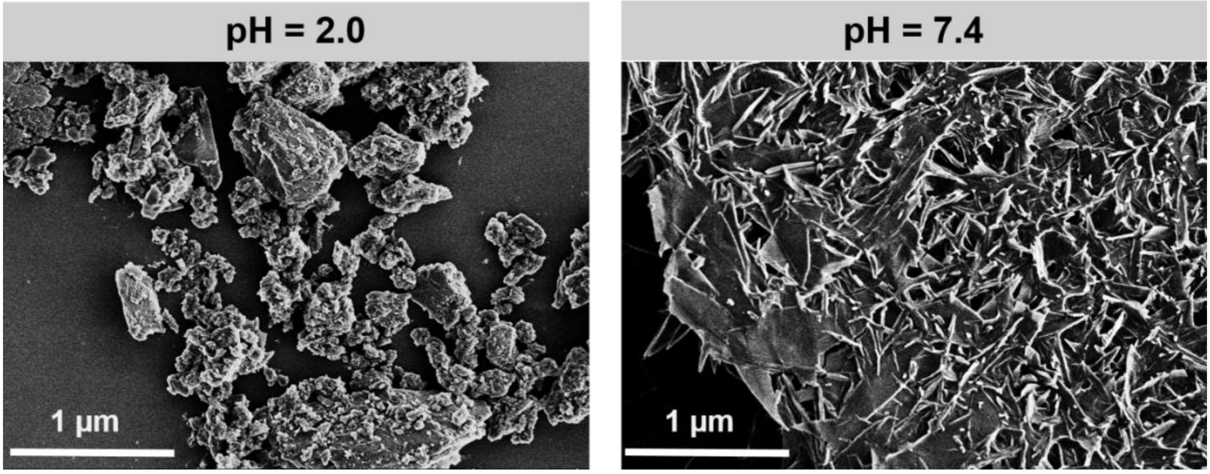

Figure S10. SEM images of CAMM after treatment with simulated gastric fluid (SGF, pH 2.0) and simulated colonic fluid (SCF, pH 7.4) for 2 h.

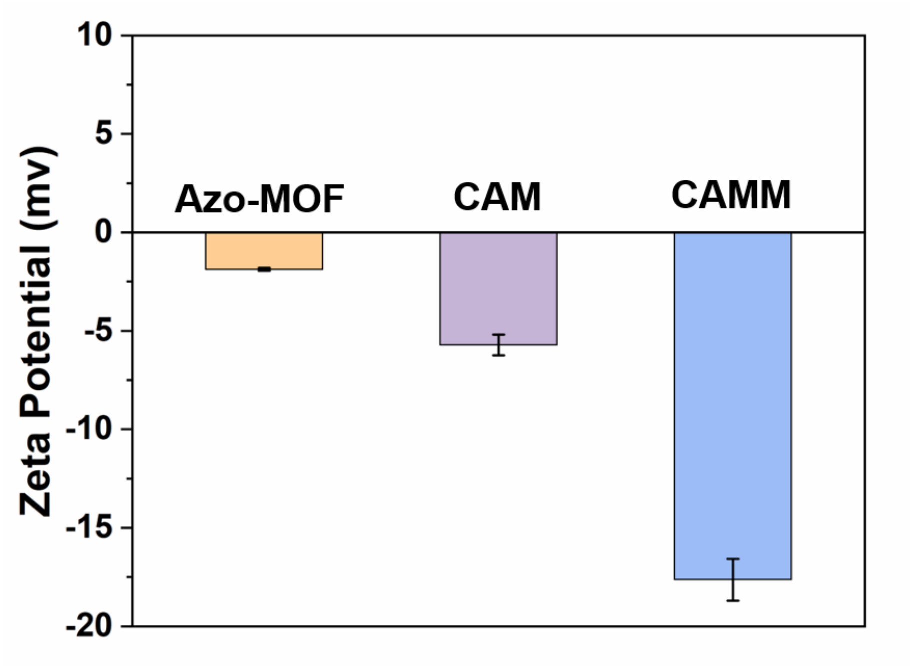

Figure S11. Zeta potential of Azo-MOF, CAM, and CAMM. Data are presented as means ± SD (*n* = 3). Source data are provided in the Source Data file.

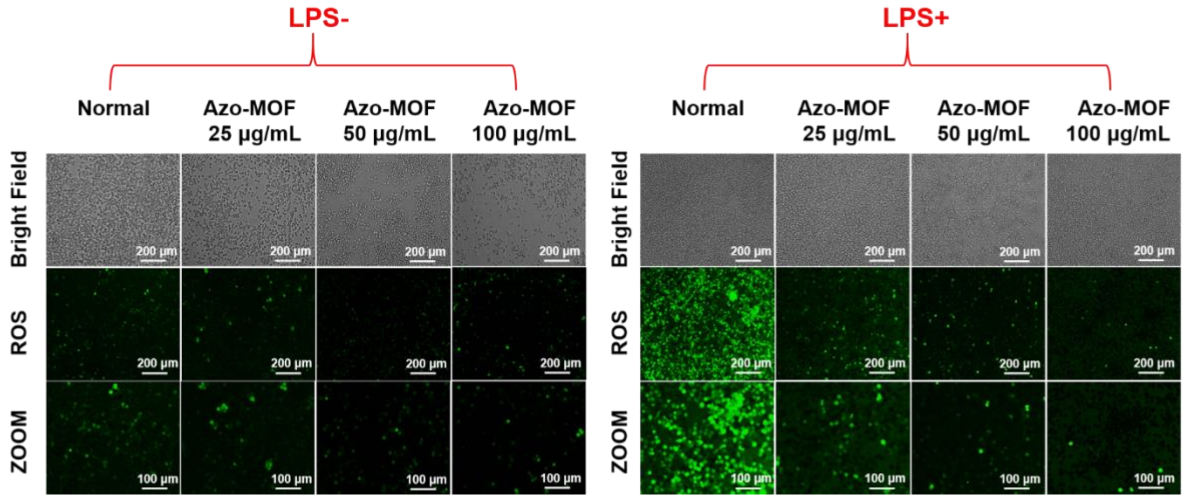

Figure S12. DCFH-DA fluorescence imaging showing ROS levels in macrophages treated with different concentrations of Azo-MOF under lipopolysaccharide (LPS)- and LPS+ conditions. Scale bars: 200 µm and 100 µm.

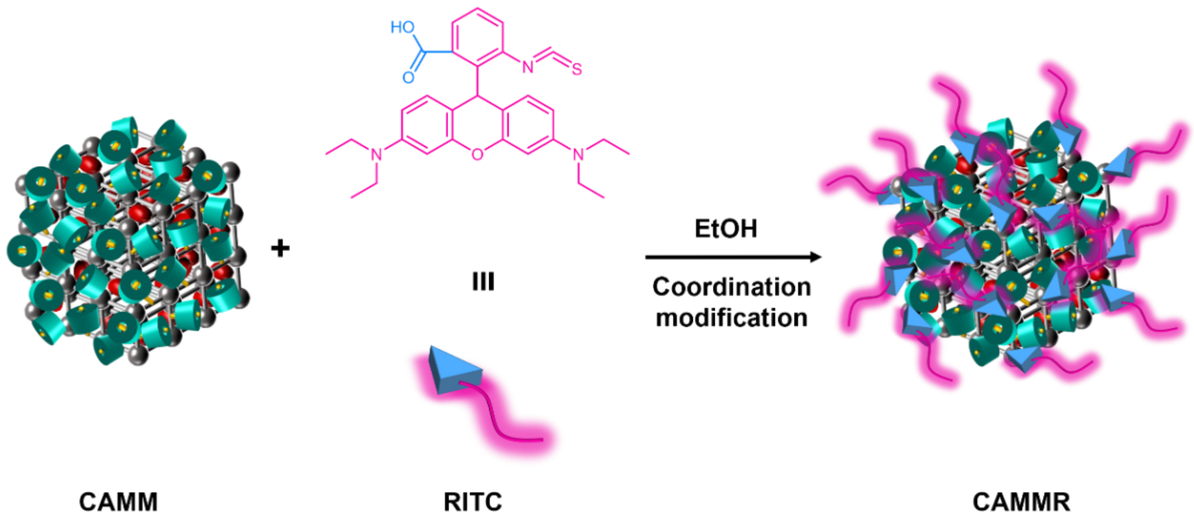

Figure S13. Synthetic scheme illustrating the fluorescent labeling mechanism for CAMMR formation through coordination modification of CAMM with RITC.

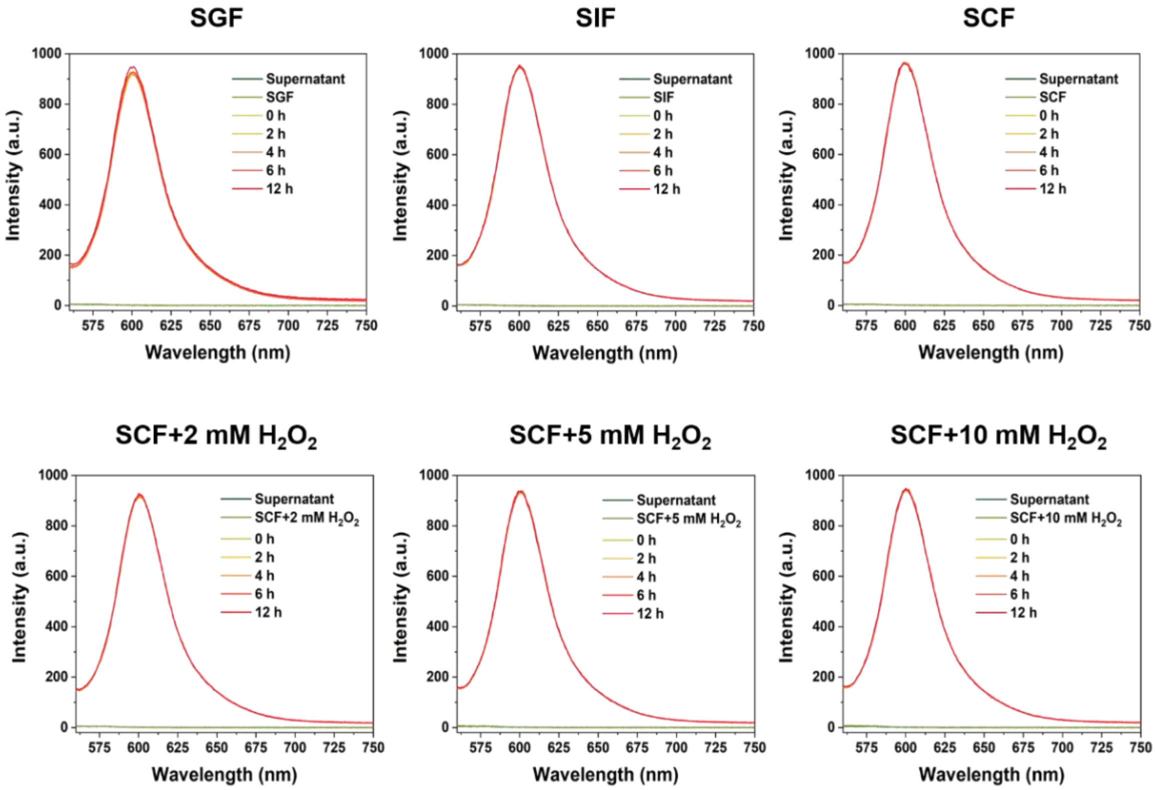

Figure S14. The fluorescence stability of CAMMR in various mediums. Source data are provided in the Source Data file.

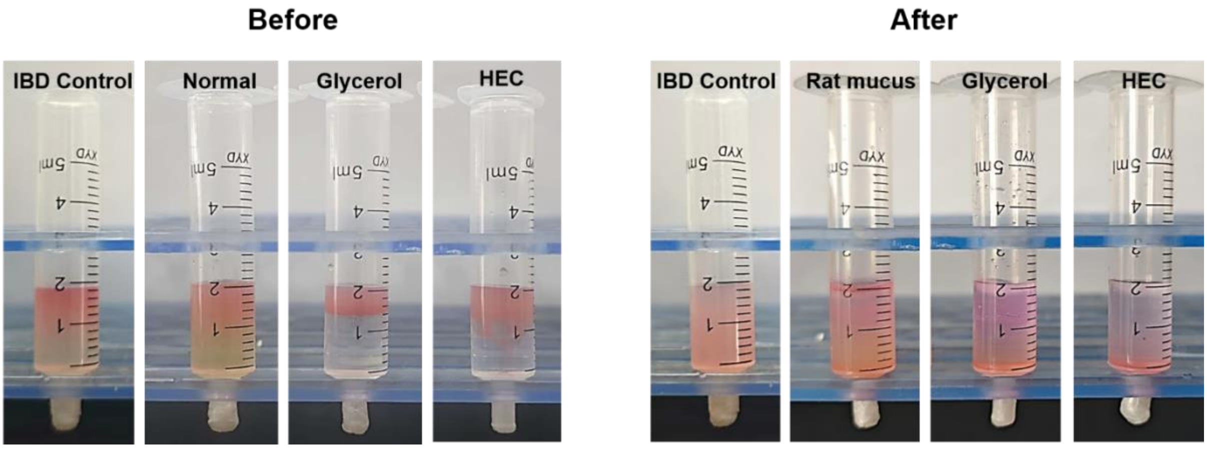

Figure S15. Diffusion images of RITC-labeled CAMMR nanomedicines in various media, including IBD rat mucus, normal rat mucus, glycerol, and HEC to simulate different physiological and pathological environments affecting drug diffusion and penetration (n = 3).

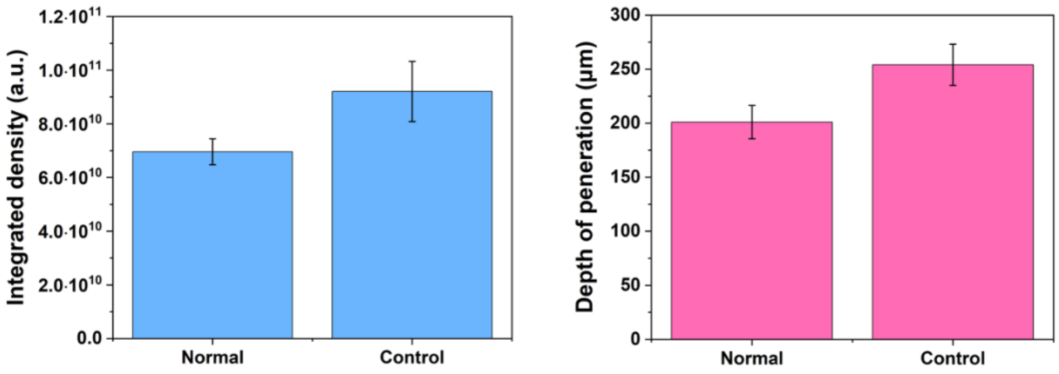

Figure S16. The fluorescence integrated density (C) and penetration depth (D) of RITC@CNS and RITC@L/D-MCNS acquired from the 2D and 3D images, respectively. Data are presented as means ± SD (n = 3). Source data are provided in the Source Data file.

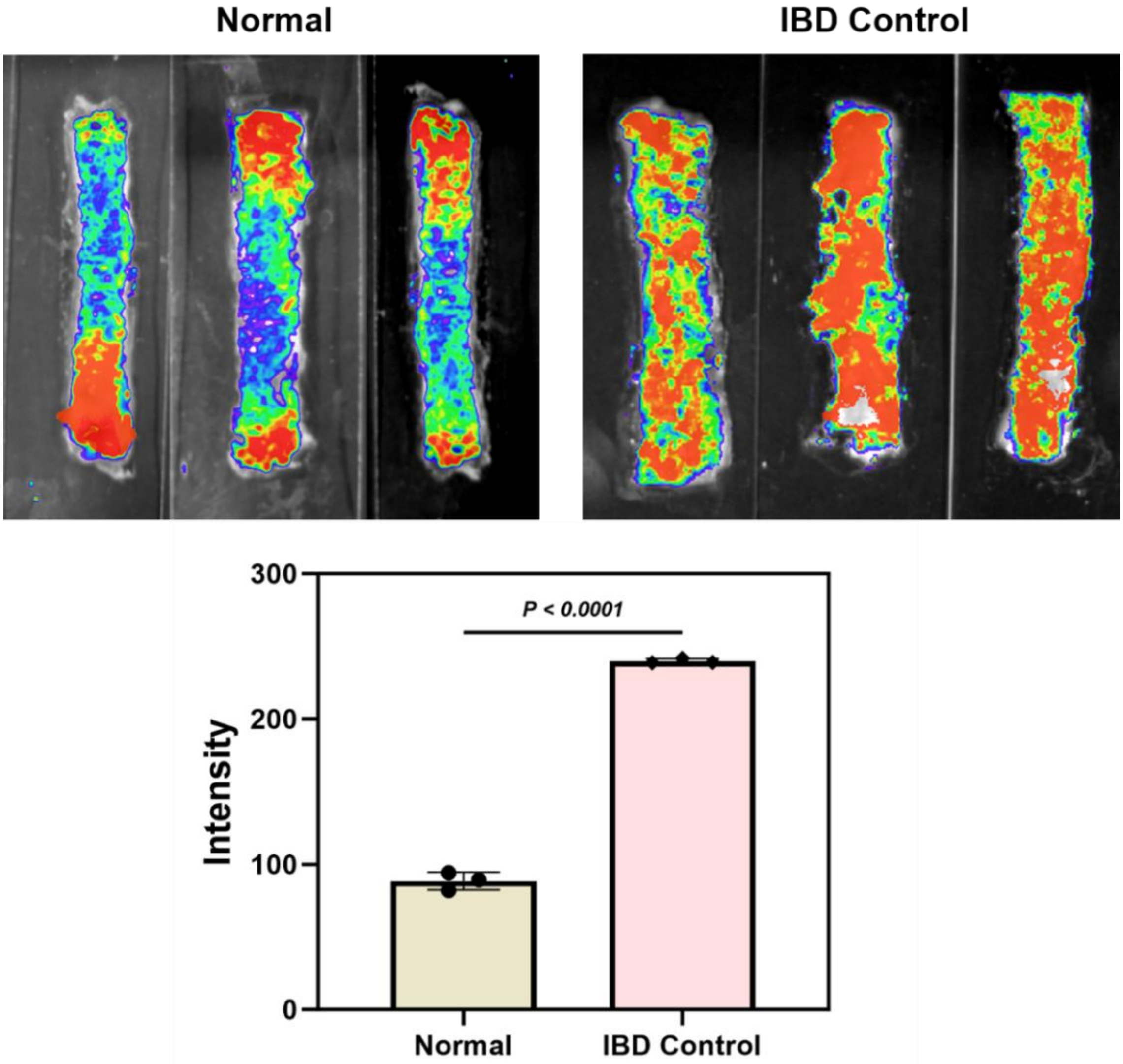

Figure S17. Appearance and fluorescence intensity of CAMMR nanoparticle retention on intestinal mucosa of healthy rats and DSS- model rat. Data are presented as means ± SD (n = 3). Source data are provided in the Source Data file.

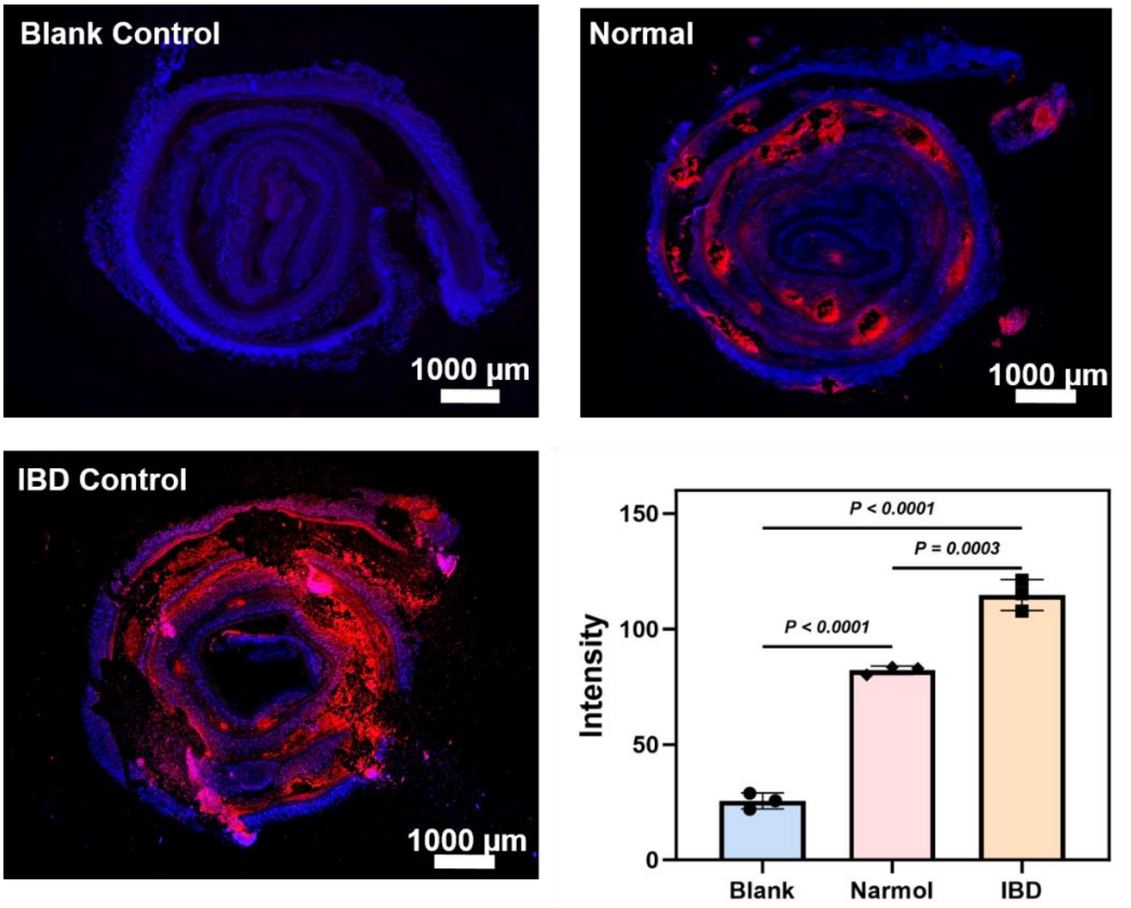

Figure S18. Swiss roll fluorescence imaging of intestinal mucosal adhesion showing blank control, CAMMR adhesion and penetration on intestinal mucosa of healthy rats and DSS-model rat, with corresponding fluorescence intensity quantification. Scale bars: 1000 μm. Data are presented as means ± SD (n = 3). Source data are provided in the Source Data file.

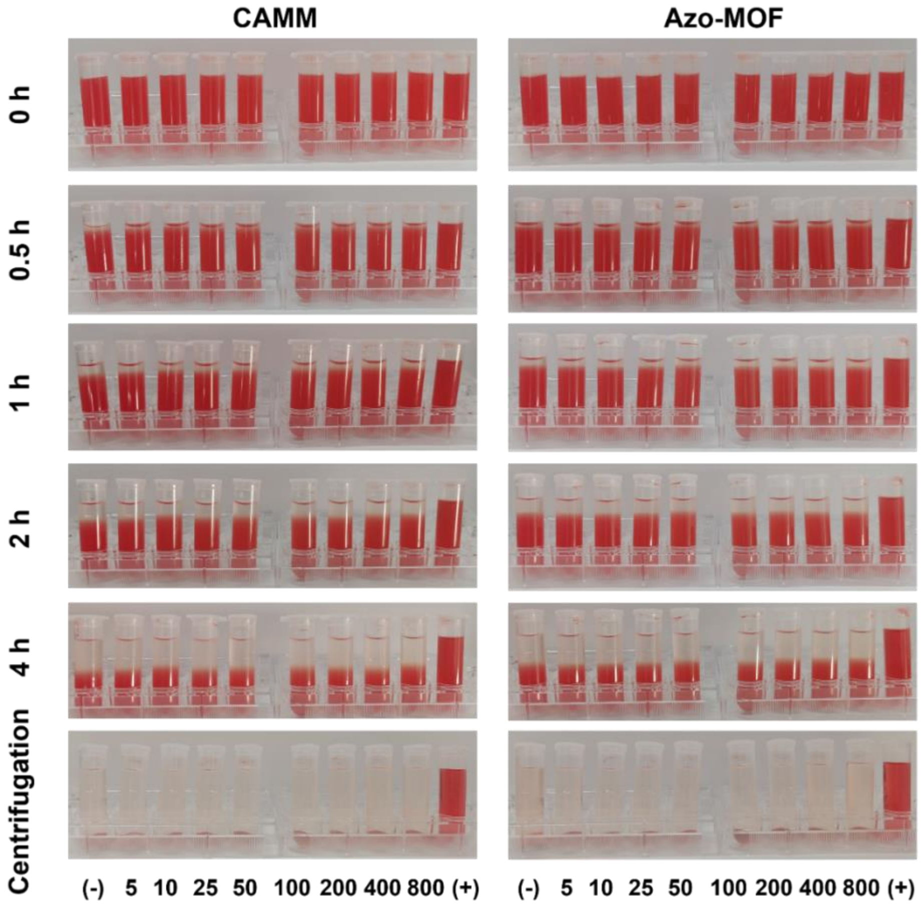

Figure S19. Blood sedimentation images and b. the hemolysis ratio of CAMM and Azo-MOF.

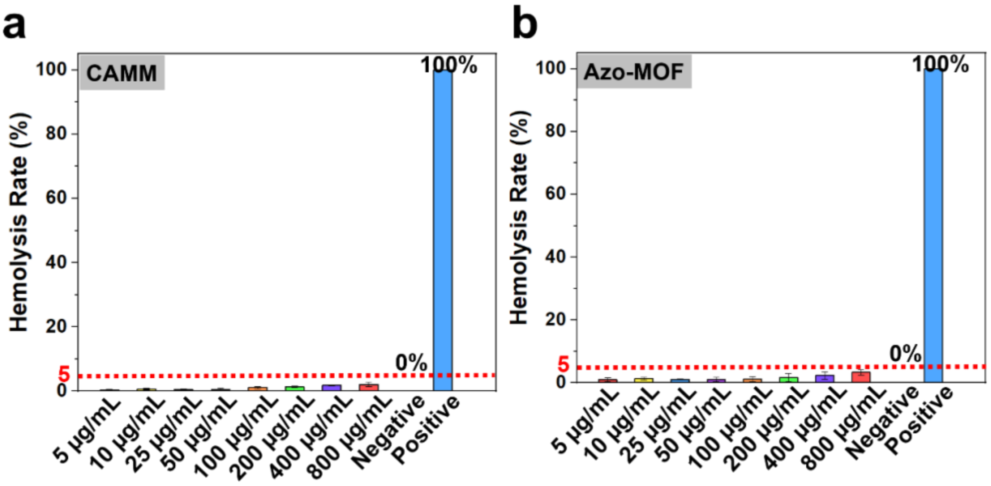

Figure S20. Hemolysis rates of **a,** CAMM and **b,** Azo-MOF at different concentrations, showing biocompatibility with hemolysis rates below 5% (indicated by red dotted line) across all tested concentrations. Data are presented as means ± SD (n = 3). Source data are provided in the Source Data file.

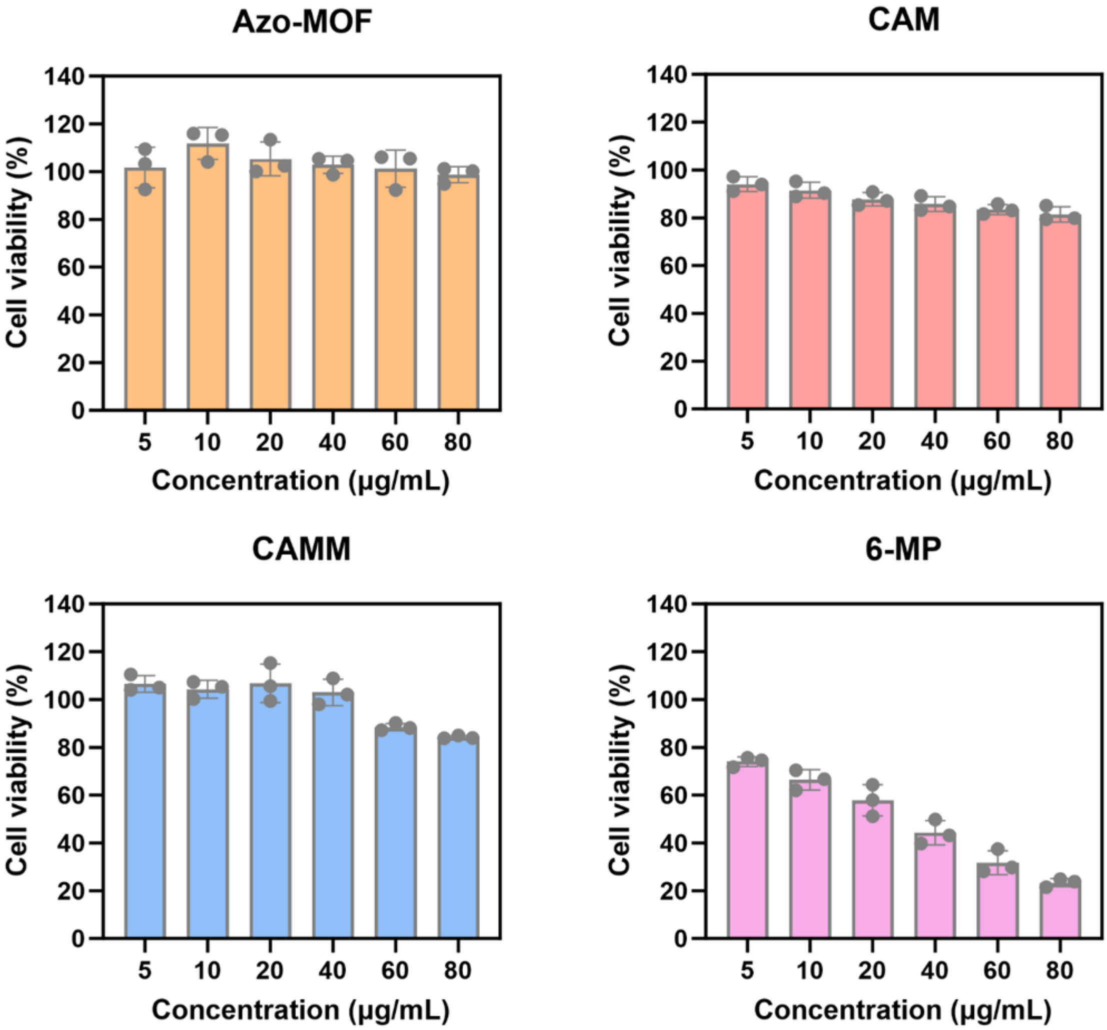

Figure S21. Cytotoxicity assay of Azo-MOF, CAM, CAMM, and 6-MP at concentrations ranging from 5 to 80 μg mL^{-1}. Data are presented as means ± SD (n = 3). Source data are provided in the Source Data file.

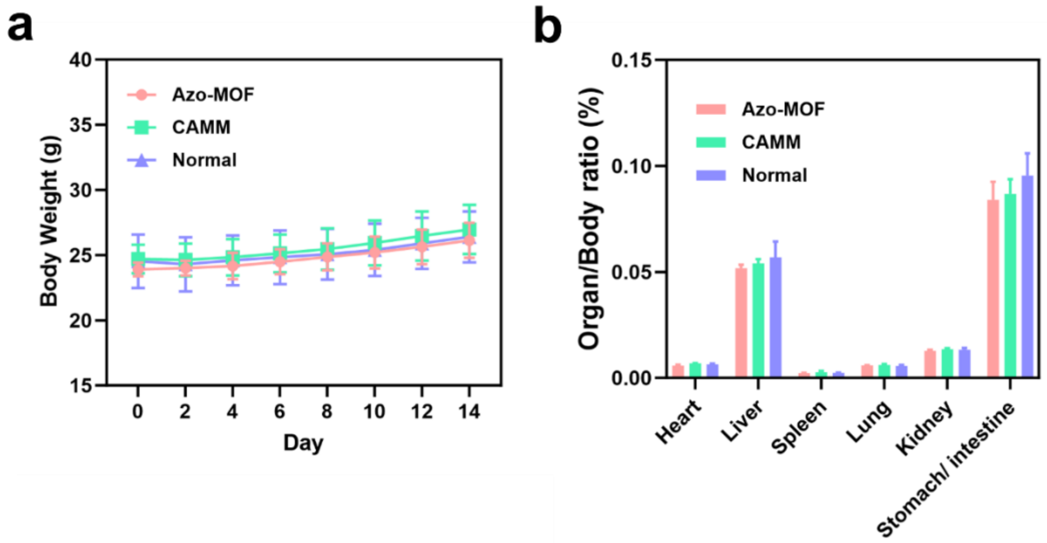

Figure S22. In vivo biocompatibility assessment showing **a,** body weight changes over 14 days and **b,** organ-to-body weight ratios for major organs in mice treated with Azo-MOF, CAMM, or normal control. *n* = 3 biologically independent animals. Data are presented as means ± SD. Source data are provided in the Source Data file.

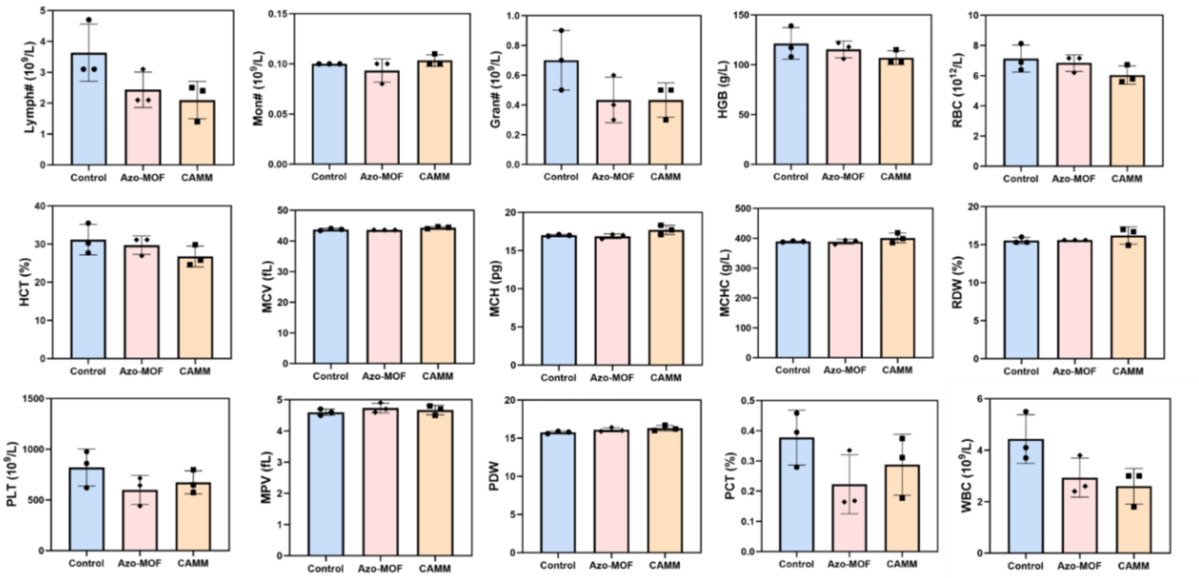

Figure S23. Hematology indexes of mice after exposure on CAMM for 14 days. n = 3 biologically independent animals. Data are presented as means ± SD. Source data are provided in the Source Data file.

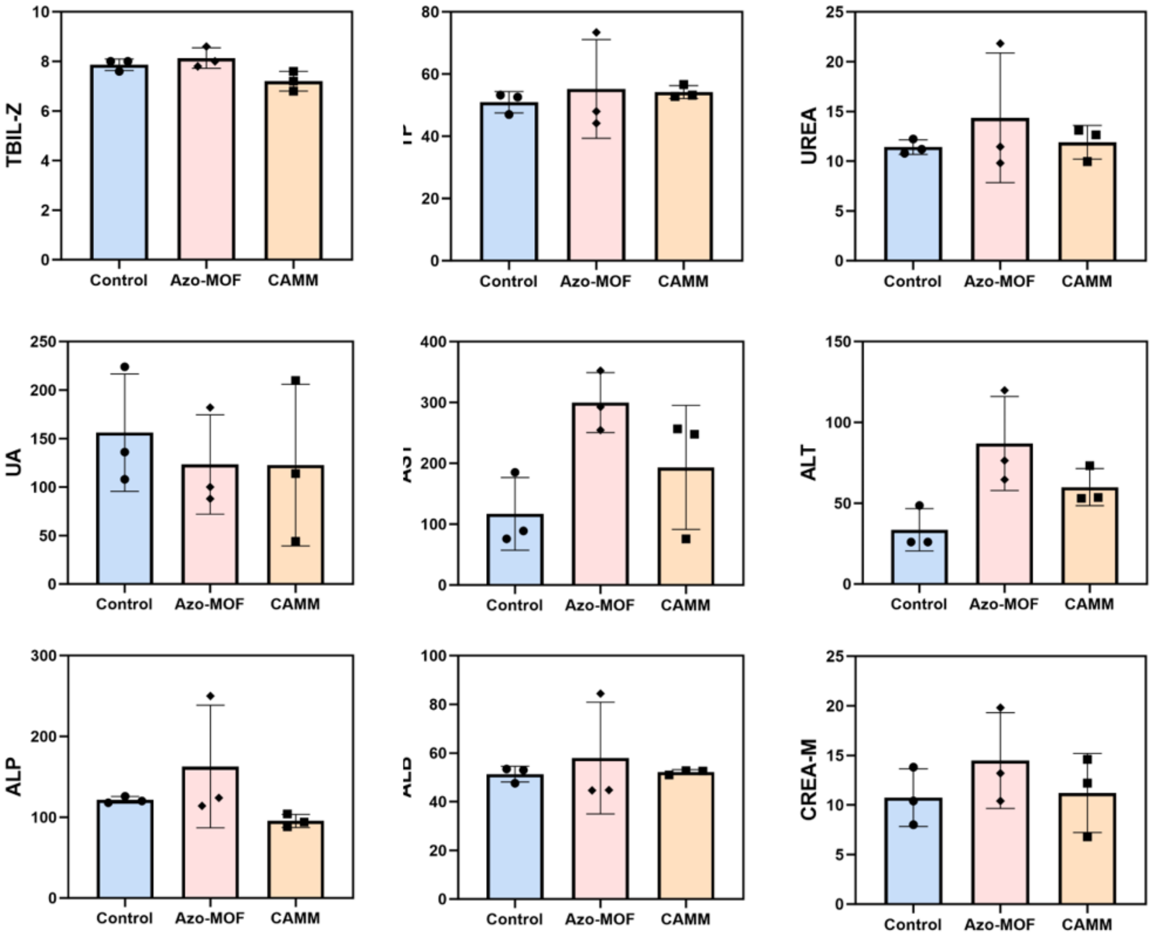

Figure S24. Biochemical indexes of mice after exposure on PEI-L/D-TA@MON for 14 days. $n = 3$ biologically independent animals. Data are presented as means ± SD. Source data are provided in the Source Data file.

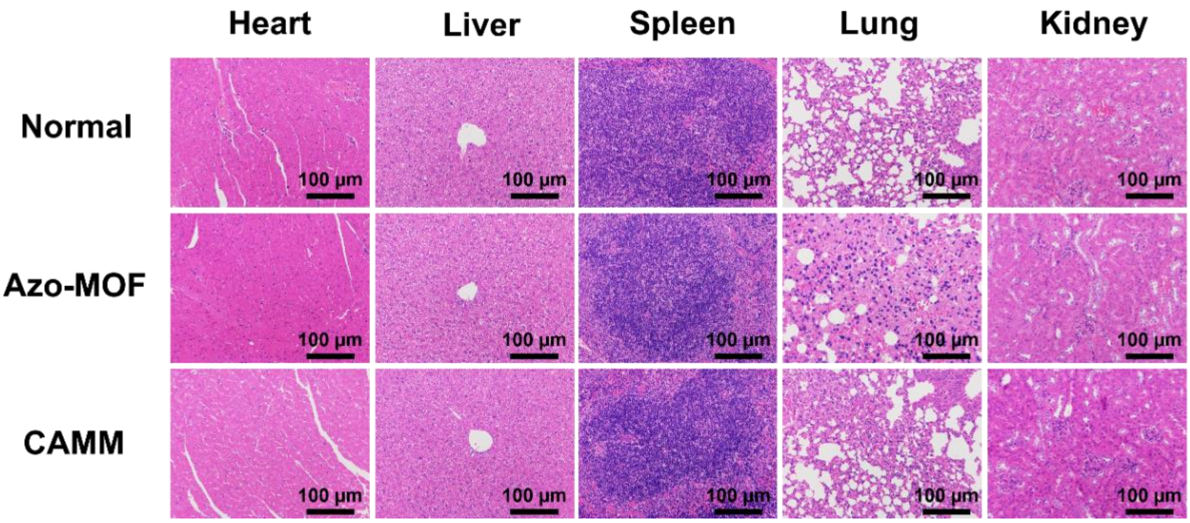

Figure S25. Histopathological examination on the main organs of mice after exposure on CAMM for 14 days. *n* = 3 biologically independent animals.

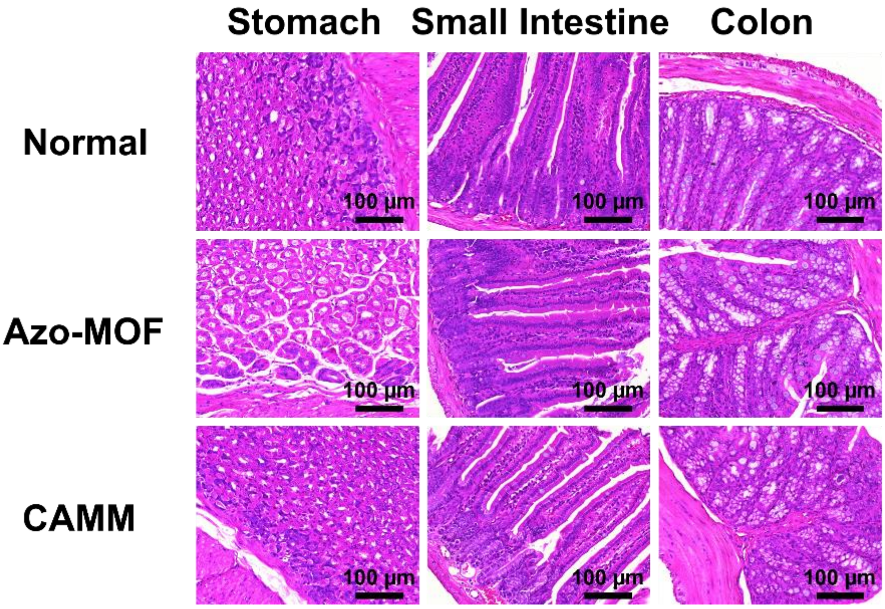

Figure S26. Histopathological examination on the GIT of mice after exposure on CAMM for 14 days. *n* = 3 biologically independent animals.

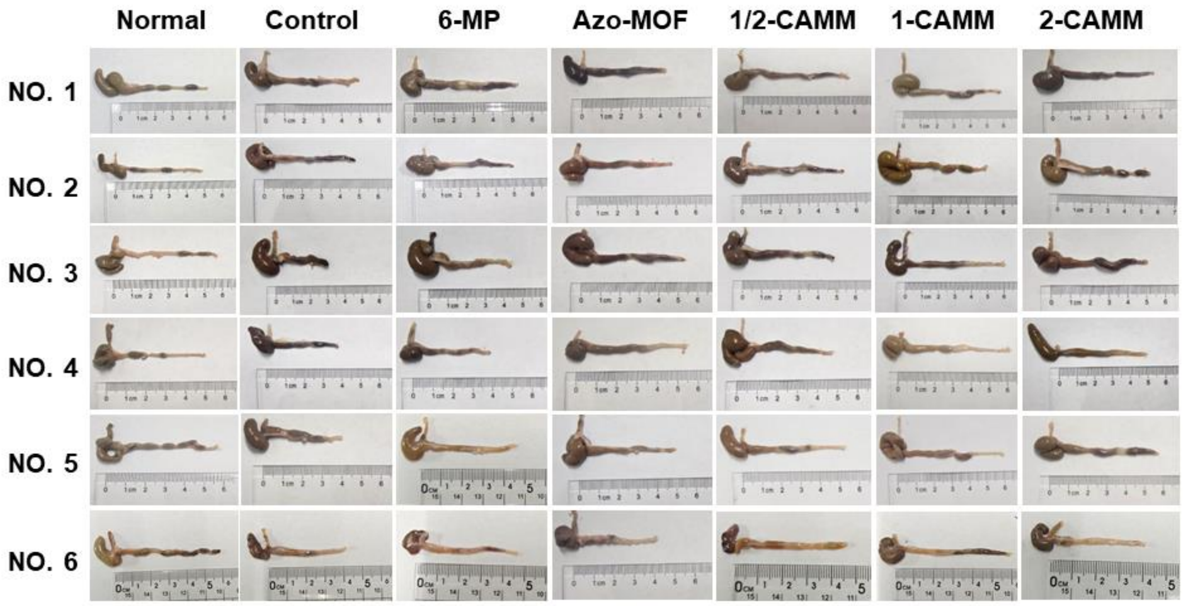

Figure S27. Colon images of DSS-model mice received prophylactic therapy with CAMM, *n* = 6 biologically independent animals.

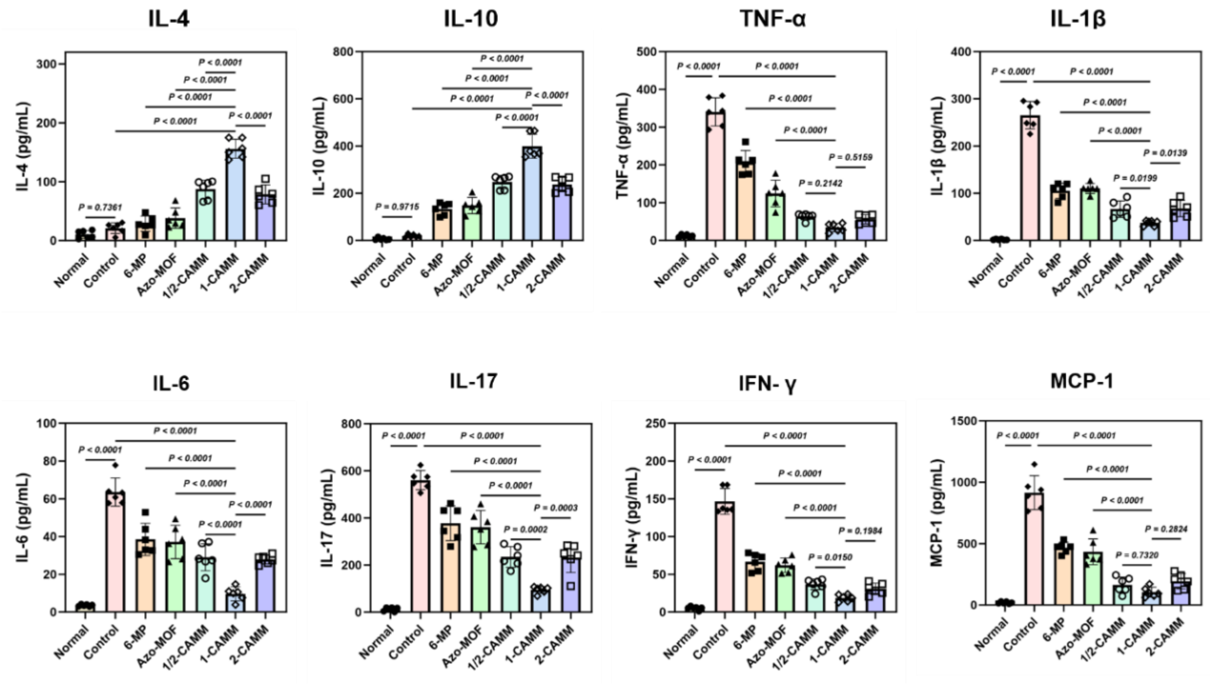

Figure S28. Cytokine levels in different treatment groups showing inflammatory markers IL-4, IL-10, TNF-α, IL-1β, IL-6, IL-17, IFN-γ, and MCP-1. n = 6 biologically independent animals. Statistical significance indicated by P values by two-tailed Student's t-test. Data are presented as means ± SD. Source data are provided in the Source Data file.

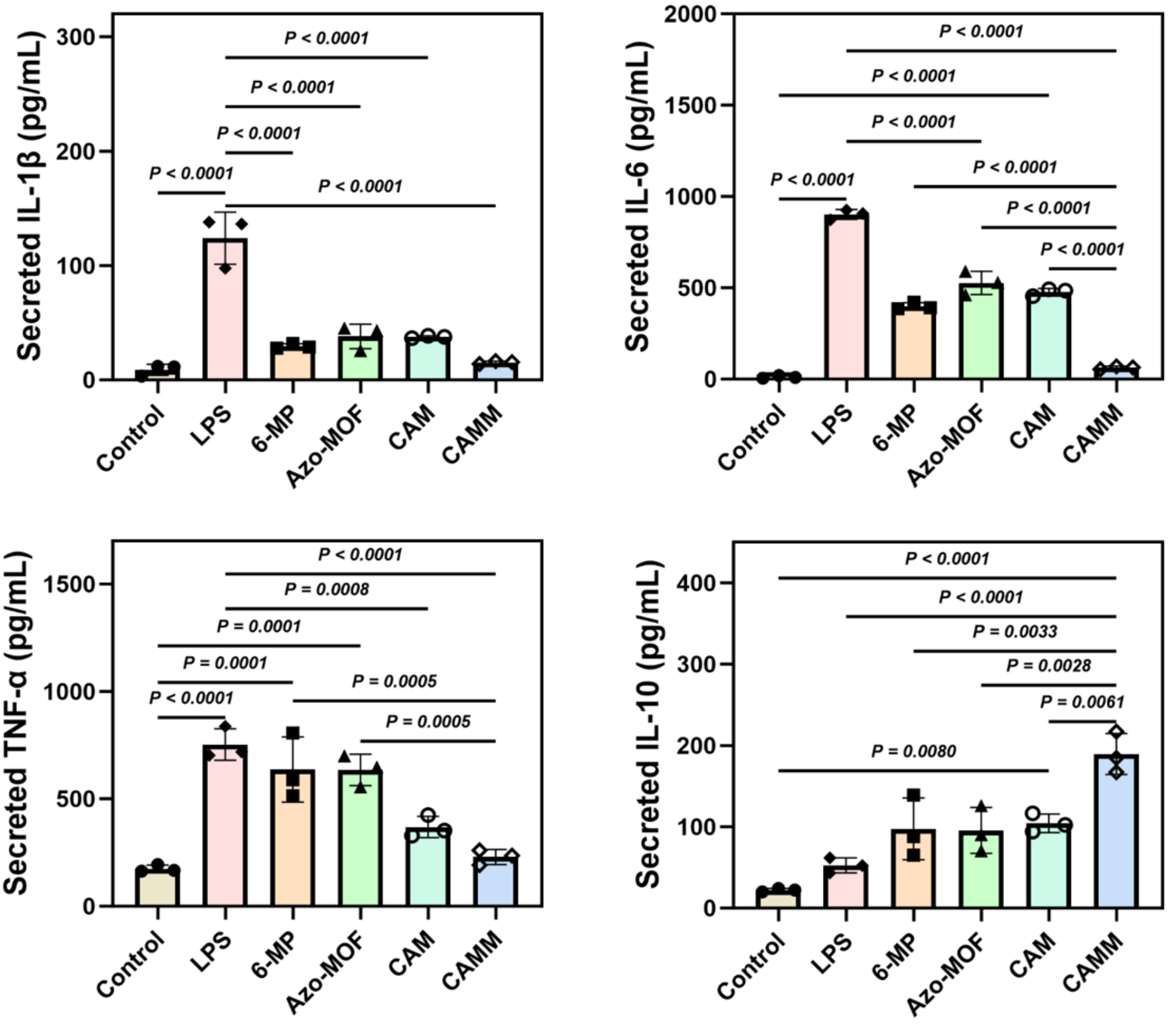

Figure S29. In vitro anti-inflammatory effects of CAMM in LPS-stimulated RAW 264.7 macrophages. ELISA quantification of secreted pro-inflammatory cytokines (IL-1β, IL-6, TNF-α) and anti-inflammatory cytokine (IL-10) in cell culture supernatants. $n = 3$ independent experiments. Statistical significance indicated by P values by two-tailed Student's t-test. Data are presented as means ± SD. Source data are provided in the Source Data file.

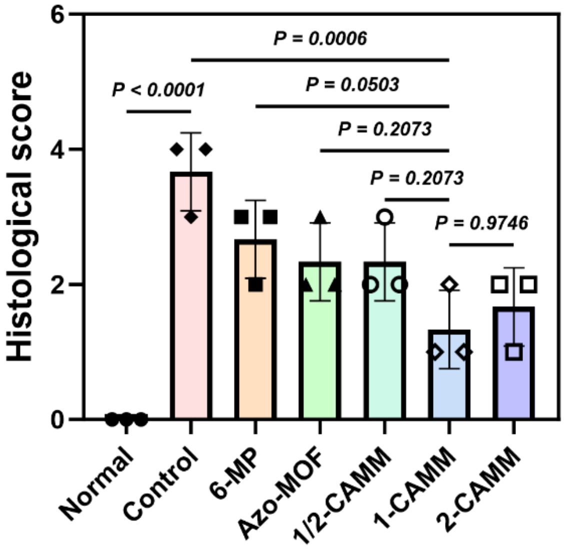

Figure S30. Histological scores of colonic tissues from DSS-model mice treated with different nanomedicines. $n = 3$ biologically independent animals. Statistical significance indicated by P values by two-tailed Student's t-test. Data are presented as means ± SD. Source data are provided in the Source Data file.

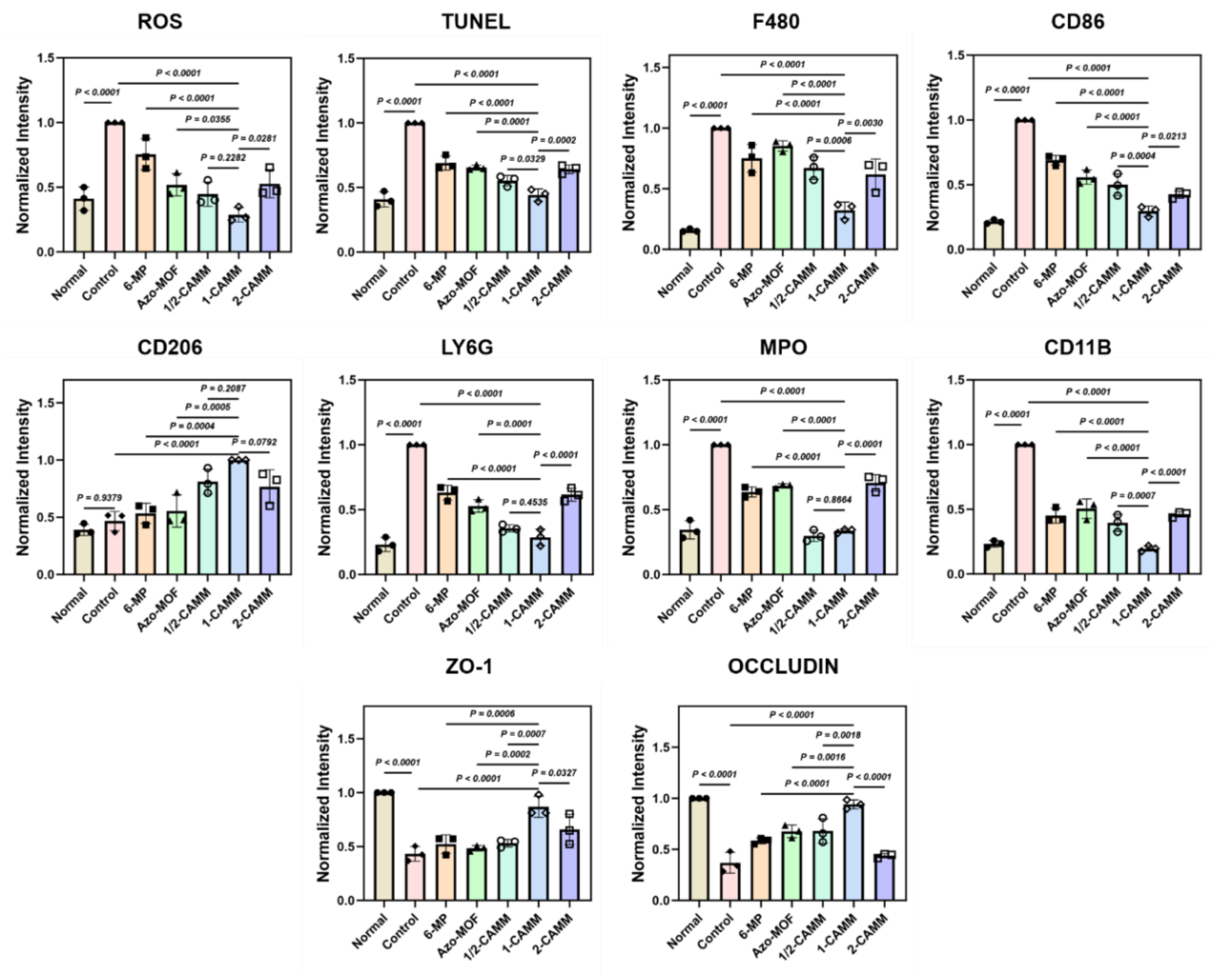

Figure S31. Semiquantitative fluorescence analysis of immunofluorescence images from DSS-model mice receiving prophylactic therapy with nanomedicines. $n = 3$ biologically independent animals. Statistical significance indicated by P values by two-tailed Student's t-test. Data are presented as means ± SD. Source data are provided in the Source Data file.

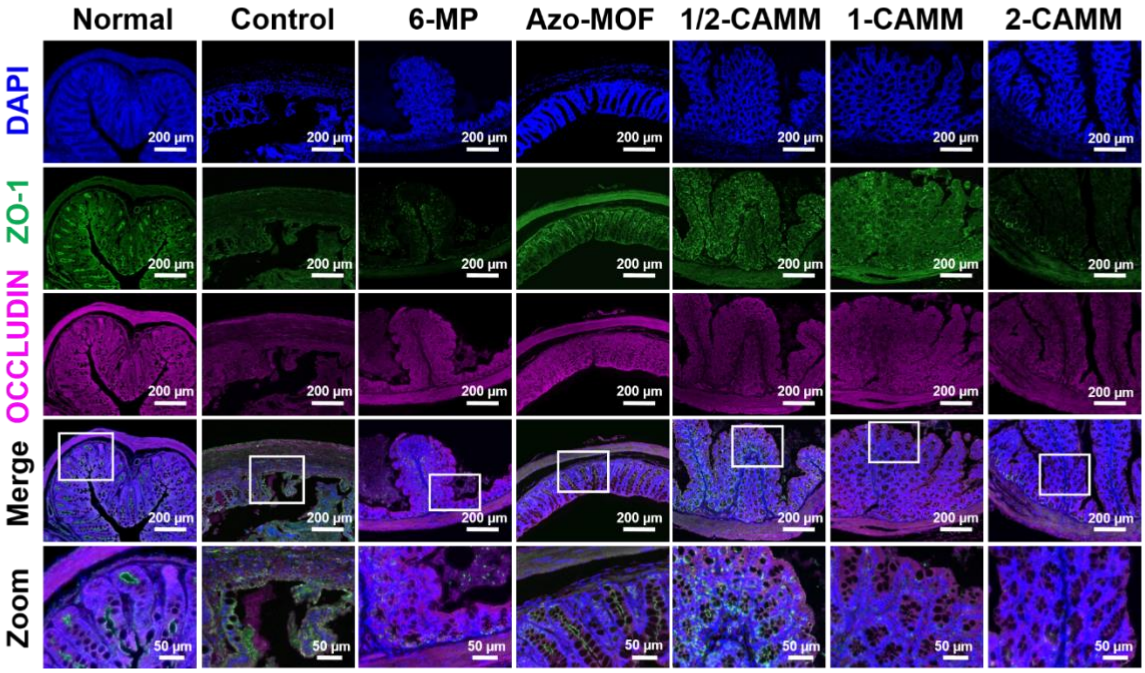

Figure S32. ZO-1 and Occludin staining (blue: nucleus stained with DAPI, green: ZO-1, purple: Occludin) on the colon of mice after exposure on CAMM for 14 days to indicate the integrity on intestinal barrier. *n* = 3 biologically independent animals.

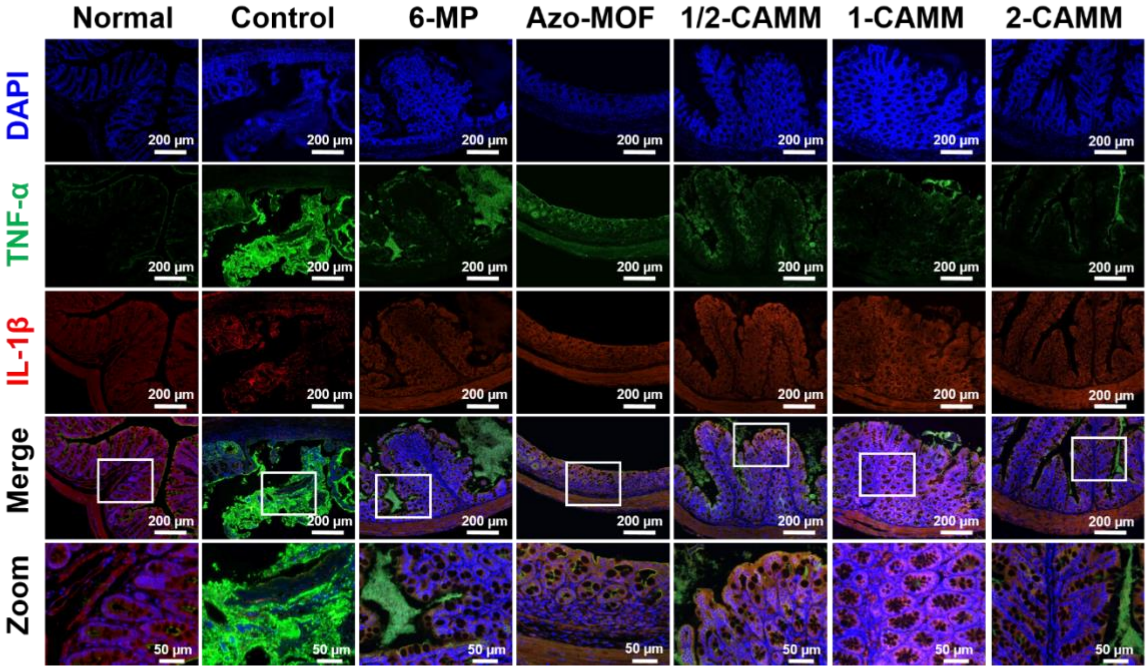

Figure S33. TNF-α and IL-1β immunofluorescence staining (blue: nucleus stained with DAPI, green: TNF-α, red: IL-1β, merged and zoomed images shown) in colon tissues of DSS-model mice treated with different nanomedicines. n = 3 biologically independent animals.

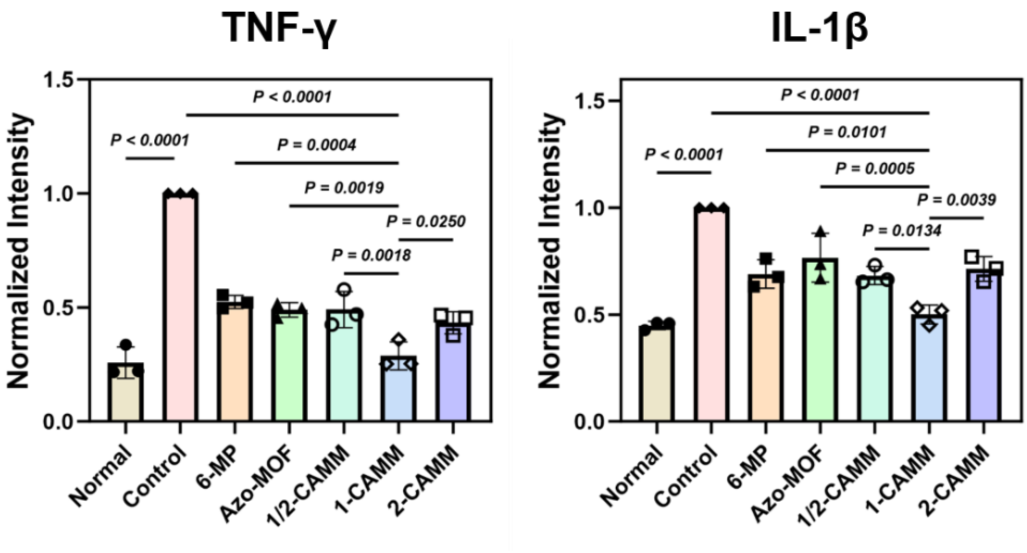

Figure S34. TNF-γ and IL-1β cytokine levels in homogenized colonic tissues of DSS-model mice treated with nanomedicines. *n* = 3 biologically independent animals. Statistical significance indicated by *P* values by two-tailed Student's t-test. Data are presented as means ± SD. Source data are provided in the Source Data file.

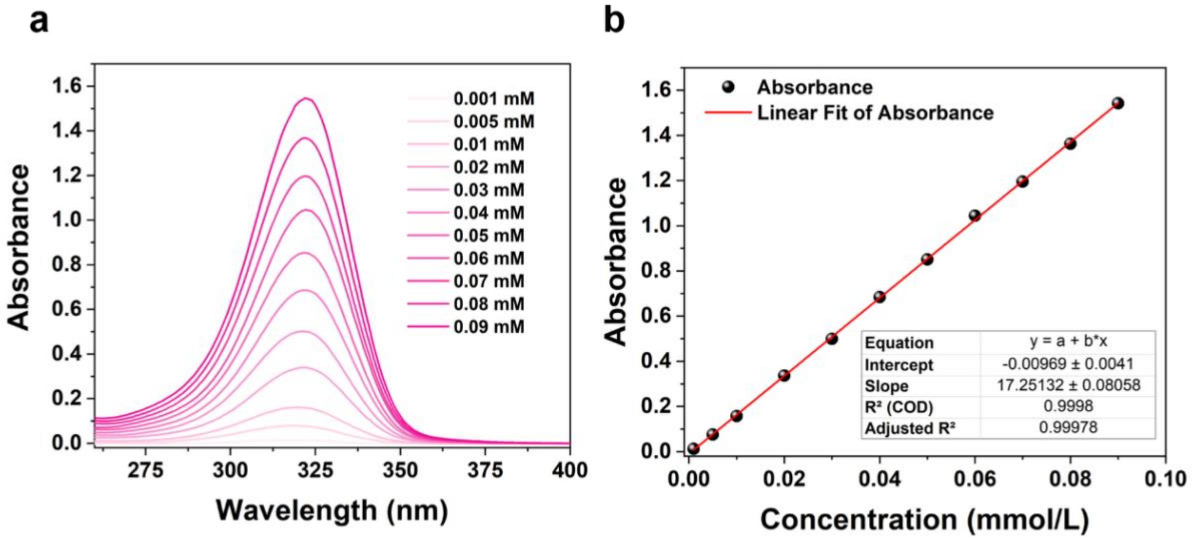

Figure S35. a, UV-Vis absorption spectra of 6-MP at different concentrations (0.001-0.09 mM) in aqueous solution. **b,** Standard calibration curve of 6-MP showing linear relationship between concentration and absorbance with equation y = 17.26132x + 0.08058 (R^2 = 0.99978). Source data are provided in the Source Data file.

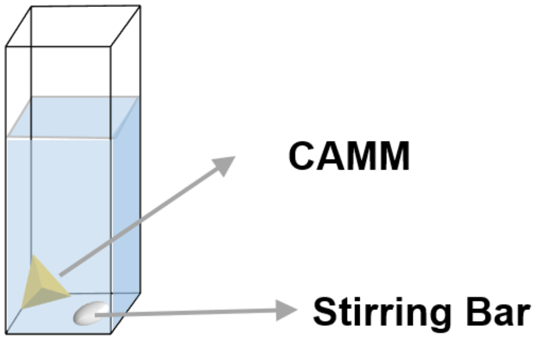

Figure S36. Schematic diagram of the experimental setup for CAMM controlled release studies.

Table S1. Crystal data and structure refinement parameters for Azo-MOF

Parameters	Azo-MOF
CCDC number	2421494
Empirical formula	$C_{84}H_{66}N_8O_{18}Zn_5$
Formula weight	1958.56
Temperature (K)	100.00(10)
Crystal system	triclinic
Space group	$P\bar{1}$(2)
a (Å)	6.2953(1)
b (Å)	19.2674(3)
c (Å)	22.2993(4)
α (°)	80.982(2)
β (°)	85.614(2)
γ (°)	85.050(1)
V (Å^3)	2655.92(8)
Z	1
R_{int}	0.0375
R_{sigma}	0.0487
ρ_{calc} (g cm^{-3})	1.225
μ (mm^{-1})	1.176
F(000)	1010
Reflections collected	37900
Independent reflections	12104
Goodness-of-fit on F^2	1.055
R [$I \geq 2\sigma(I)$, all data]	0.0543
R_w [$I \geq 2\sigma(I)$, all data]	0.1330

Table S2. Disease activity index = (Weight loss + Stool consistency + Rectal bleeding)/3

Score	Weight loss (%)	Stool consistency	Rectal bleeding
0	<1	Normal	Normal
1	1-5	N/A	N/A
2	5-10	Loose stool	Fecal occult blood
3	10-15	N/A	N/A
4	>15	Diarrhea	Obvious bleeding

Table S3. Histologic scores for colon

Score	Ulceration	Epithelial damage	Inflammatory infiltration	Lymph node
0	0	Normal	No infiltrate	0
1	1	Loss of goblet cells	Infiltrate around crypt basis	1
2	2	Large area loss of goblet cells	Infiltrate reaching the lamina muscular mucosae	2
3	3	Loss of crypts	Extensive infiltrate reaching the lamina muscular mucosae with thickening of the mucosa	3
4	>3	Large area loss of crypts	Submucosal infiltration	>3

Table S4. Four-parameter logistic function curve: y=A2+(A1-A2)/(1+x/x0)

	TNF-α	IL-1β	IL-4	IL-6	IL-10	IL-17A	INF-γ	MCP-1
A1	0.05689 ± 0.03161	0.18041 ± 0.02125	0.05393 ± 0.00944	0.04081 ± 0.00894	0.04241 ± 0.00389	0.07385 ± 0.00773	0.05579 ± 0.00965	0.07463 ± 0.03077
A2	4.17476 ± 0.49254	6.02981 ± 0.63591	3.04256 ± 0.24902	6.17201 ± 0.37517	6.83008 ± 0.72769	5.70412 ± 0.50964	7.09461 ± 0.72351	1.13686 ± 0.0936
x0	315.10854 ± 66.59727	534.90154 ± 93.39557	1675.86542 ± 252.57678	2442.68307 ± 273.02099	3029.77426 ± 511.47728	2695.70357 ± 394.59863	3421.29506 ± 641.18149	729.03559 ± 79.3888
p	1.19224 ± 0.11596	1.1727 ± 0.07151	1.09927 ± 0.05785	1.00312 ± 0.02768	0.97308 ± 0.02188	1.10826 ± 0.03944	0.94413 ± 0.03327	3.09206 ± 0.79836

Table S5. Baseline characteristics of antibodies used in IF, IHC

Antibody	Company	Catalog Number	Clone Name	Lot Number	Dilution fold	Application
Anti-MPO	abcam	ab208670	EPR20257	1041028-54	1:1000	IHC, IF
Anti-LY6G	abcam	ab238132	EPR22909-135	1073751-11	1:4000	IHC, IF
Anti-ZO-1	abcam	ab221547	EPR19945-296	N/A	1:3000	IHC, IF
Anti-occludin	abcam	ab216327	EPR20992	N/A	1:800	IHC, IF
Anti-F480	CST	70076	D2S9R	9	1:600	IHC, IF
Anti-TNF-α	abcam	ab307164	RM1005	N/A	1:800	IHC, IF
Anti-IL-1β	abcam	ab283818	RM1009	N/A	1:800	IHC, IF
Anti-CD86	CST	19589	E5W6H	N/A	1:400	IHC, IF
Anti-CD206	CST	24595	E6T5J	N/A	1:1000	IHC, IF
Anti-CD11b	ABclonal	A1581	N/A	N/A	1:100	IHC, IF

Table S6. Baseline characteristics of antibodies used in animal experiments

Inflammatory Factors	Company	Catalog No
Mouse IFN-γ	Elabscience	E-MSEL-M0007
Mouse IL-1β	Elabscience	E-MSEL-M0003
Mouse IL-4	Elabscience	E-MSEL-M0008
Mouse IL-6	Elabscience	E-MSEL-M0001
Mouse IL-10	Elabscience	E-MSEL-M0031
Mouse IL-17A	Elabscience	E-MSEL-M0006
Mouse MCP-1	Elabscience	E-MSEL-M0012
Mouse TNF-α	Elabscience	E-MSEL-M0002
