# Supplementary material for: Construction of a MOF‐Based Snap‐Top Delivery Nanosystem for Powerful Dual‐Responsive Synergistic Colitis Treatment
Source: Adv Sci (Weinh). 2026 Apr 2;13(36):e24174. doi: 10.1002/advs.202524174 (PMC13317788; doi:10.1002/advs.202524174)

checkCIF/PLATON report

Structure factors have been supplied for datablock(s) 1

THIS REPORT IS FOR GUIDANCE ONLY. IF USED AS PART OF A REVIEW PROCEDURE FOR PUBLICATION, IT SHOULD NOT REPLACE THE EXPERTISE OF AN EXPERIENCED CRYSTALLOGRAPHIC REFEREE.

No syntax errors found. CIF dictionary Interpreting this report

Datablock: 1

```
Bond precision:   C-C = 0.0028 A          Wavelength=0.71073

Cell:           a=6.2953(1)        b=19.2674(3)      c=22.2993(4)
                alpha=80.982(2)    beta=85.614(2)    gamma=85.050(1)
Temperature:    100 K
```

	Calculated	Reported
Volume	2655.92(8)	2655.92(8)
Space group	P -1	P -1
Hall group	-P 1	-P 1
Moiety formula	C78 H52 N6 O16 Zn5, 2(C3 H7 N O) [+ solvent]	C78 H52 N6 O16 Zn5, 2(C3 H7 N O), 2[C3H7NO], 0.5[H2O]
Sum formula	C84 H66 N8 O18 Zn5 [+ solvent]	C90 H90 N10 O20 Zn5
Mr	1802.40	1958.56
Dx,g cm-3	1.127	1.225
Z	1	1
Mu (mm-1)	1.169	1.176
F000	920.0	1010.0
F000'	921.77	
h,k,lmax	8,26,30	8,25,29
Nref	14826	12104
Tmin,Tmax	0.858,0.889	0.540,1.000
Tmin'	0.858	

```
Correction method= # Reported T Limits: Tmin=0.540 Tmax=1.000
AbsCorr = MULTI-SCAN

Data completeness= 0.816           Theta(max)= 29.503
```

R(reflections)= 0.0422(9719)

wR2(reflections)= 0.1330(12104)

S = 1.055

Npar= 586

The following ALERTS were generated. Each ALERT has the format
test-name_ALERT_alert-type_alert-level.
Click on the hyperlinks for more details of the test.

Alert level B

PLAT220_ALERT_2_B NonSolvent Resd 1 C Ueq(max)/Ueq(min) Range 7.3 Ratio

Author Response: This alert is generated because there is a large amount of disorder in the structure. Dynamically disordered side groups may exhibit such unusual ratios.

PLAT420_ALERT_2_B D-H Bond Without Acceptor O2 --H2 . Please Check

Author Response: The solvent molecules are highly disordered, which supposed to form hydrogen bond with water molecules have been removed by the SQUEEZE routine in the PLATON software package.

Alert level C

PLAT222_ALERT_3_C NonSolvent Resd 1 H Uiso(max)/Uiso(min) Range 5.7 Ratio
PLAT250_ALERT_2_C Large U3/U1 Ratio for Average U(i,j) Tensor .... 2.7 Note
PLAT911_ALERT_3_C Missing FCF Refl Between Thmin & STh/L= 0.600 13 Report
PLAT977_ALERT_2_C Check Negative Difference Density on H1 . -0.33 eA-3

Alert level G

```
FORMU01_ALERT_1_G  There is a discrepancy between the atom counts in the
            _chemical_formula_sum and _chemical_formula_moiety. This is
            usually due to the moiety formula being in the wrong format.
            Atom count from _chemical_formula_sum:   C90 H90 N10 O20 Zn5
            Atom count from _chemical_formula_moiety:C90 H80 N10 O20 Zn5
FORMU01_ALERT_2_G  There is a discrepancy between the atom counts in the
            _chemical_formula_sum and the formula from the _atom_site* data.
            Atom count from _chemical_formula_sum:C90 H90 N10 O20 Zn5
            Atom count from the _atom_site data:  C84 H66 N8 O18 Zn5
CELLZ01_ALERT_1_G Difference between formula and atom_site contents detected.
CELLZ01_ALERT_1_G ALERT: Large difference may be due to a
            symmetry error - see SYMMG tests
           From the CIF: _cell_formula_units_Z    1
           From the CIF: _chemical_formula_sum  C90 H90 N10 O20 Zn5
           TEST: Compare cell contents of formula and atom_site data

           atom    Z*formula  cif sites diff
           C         90.00     84.00    6.00
           H         90.00     66.00   24.00
```

```
          N         10.00      8.00    2.00
          O         20.00     18.00    2.00
          Zn         5.00      5.00    0.00
PLAT003_ALERT_2_G Number of Uiso or Uij Restrained non-H Atoms ...         36 Report
PLAT004_ALERT_5_G Polymeric Structure Found with Maximum Dimension          3 Info
PLAT007_ALERT_5_G Number of Unrefined Donor-H Atoms .............           2 Report
PLAT041_ALERT_1_G Calc. and Reported SumFormula    Strings  Differ     Please Check
PLAT178_ALERT_4_G The CIF-Embedded .res File Contains SIMU Records          3 Report
PLAT186_ALERT_4_G The CIF-Embedded .res File Contains ISOR Records          2 Report
PLAT188_ALERT_3_G A Non-default SIMU Restraint Value has been used     0.0100 Report
PLAT188_ALERT_3_G A Non-default SIMU Restraint Value has been used     0.0050 Report
PLAT188_ALERT_3_G A Non-default SIMU Restraint Value has been used     0.0050 Report
PLAT300_ALERT_4_G Atom Site Occupancy of N1         Constrained at        0.5 Check
PLAT300_ALERT_4_G Atom Site Occupancy of N2         Constrained at        0.5 Check
PLAT300_ALERT_4_G Atom Site Occupancy of C11        Constrained at        0.5 Check
PLAT300_ALERT_4_G Atom Site Occupancy of C12        Constrained at        0.5 Check
PLAT300_ALERT_4_G Atom Site Occupancy of C13        Constrained at        0.5 Check
PLAT300_ALERT_4_G Atom Site Occupancy of C14        Constrained at        0.5 Check
PLAT300_ALERT_4_G Atom Site Occupancy of C15        Constrained at        0.5 Check
PLAT300_ALERT_4_G Atom Site Occupancy of C16        Constrained at        0.5 Check
PLAT300_ALERT_4_G Atom Site Occupancy of H10        Constrained at        0.5 Check
PLAT300_ALERT_4_G Atom Site Occupancy of H12        Constrained at        0.5 Check
PLAT300_ALERT_4_G Atom Site Occupancy of H13        Constrained at        0.5 Check
PLAT300_ALERT_4_G Atom Site Occupancy of H14        Constrained at        0.5 Check
PLAT300_ALERT_4_G Atom Site Occupancy of H15        Constrained at        0.5 Check
PLAT300_ALERT_4_G Atom Site Occupancy of H16        Constrained at        0.5 Check
PLAT301_ALERT_3_G Main Residue  Disorder .............(Resd  1  )        23% Note
PLAT302_ALERT_4_G Anion/Solvent/Minor-Residue Disorder (Resd  2  )        80% Note
PLAT606_ALERT_4_G Solvent Accessible VOID(S) in Structure ........          ! Info
PLAT794_ALERT_5_G Tentative Bond Valency for Zn1       (II)      .       1.99 Info
PLAT794_ALERT_5_G Tentative Bond Valency for Zn2       (II)      .       2.09 Info
PLAT794_ALERT_5_G Tentative Bond Valency for Zn3       (II)      .       2.07 Info
PLAT811_ALERT_5_G No ADDSYM Analysis: Too Many Excluded Atoms ....          ! Info
PLAT860_ALERT_3_G Number of Least-Squares Restraints ............         348 Note
PLAT868_ALERT_4_G ALERTS Due to the Use of _smtbx_masks Suppressed          ! Info
PLAT910_ALERT_3_G Missing # of FCF Reflection(s) Below Theta(Min).          3 Note
PLAT912_ALERT_4_G Missing # of FCF Reflections Above STh/L=  0.600       2622 Note
PLAT913_ALERT_3_G Missing # of Very Strong Reflections in FCF ....          2 Note
PLAT933_ALERT_2_G Number of HKL-OMIT Records in Embedded .res File         10 Note
PLAT941_ALERT_3_G Average HKL Measurement Multiplicity ...........        3.1 Low
PLAT978_ALERT_2_G Number C-C Bonds with Positive Residual Density.          1 Info
```

---

   0 **ALERT level A** = Most likely a serious problem - resolve or explain
   2 **ALERT level B** = A potentially serious problem, consider carefully
   4 **ALERT level C** = Check. Ensure it is not caused by an omission or oversight
  42 **ALERT level G** = General information/check it is not something unexpected

   4 ALERT type 1 CIF construction/syntax error, inconsistent or missing data
   8 ALERT type 2 Indicator that the structure model may be wrong or deficient
  10 ALERT type 3 Indicator that the structure quality may be low
  20 ALERT type 4 Improvement, methodology, query or suggestion
   6 ALERT type 5 Informative message, check

It is advisable to attempt to resolve as many as possible of the alerts in all categories. Often the minor alerts point to easily fixed oversights, errors and omissions in your CIF or refinement strategy, so attention to these fine details can be worthwhile. In order to resolve some of the more serious problems it may be necessary to carry out additional measurements or structure refinements. However, the purpose of your study may justify the reported deviations and the more serious of these should normally be commented upon in the discussion or experimental section of a paper or in the "special_details" fields of the CIF. checkCIF was carefully designed to identify outliers and unusual parameters, but every test has its limitations and alerts that are not important in a particular case may appear. Conversely, the absence of alerts does not guarantee there are no aspects of the results needing attention. It is up to the individual to critically assess their own results and, if necessary, seek expert advice.

Publication of your CIF in IUCr journals

A basic structural check has been run on your CIF. These basic checks will be run on all CIFs submitted for publication in IUCr journals (*Acta Crystallographica*, *Journal of Applied Crystallography*, *Journal of Synchrotron Radiation*); however, if you intend to submit to *Acta Crystallographica Section C* or *E* or *IUCrData*, you should make sure that full publication checks are run on the final version of your CIF prior to submission.

Publication of your CIF in other journals

Please refer to the *Notes for Authors* of the relevant journal for any special instructions relating to CIF submission.

PLATON version of 06/07/2023; check.def file version of 30/06/2023

Datablock 1 - ellipsoid plot

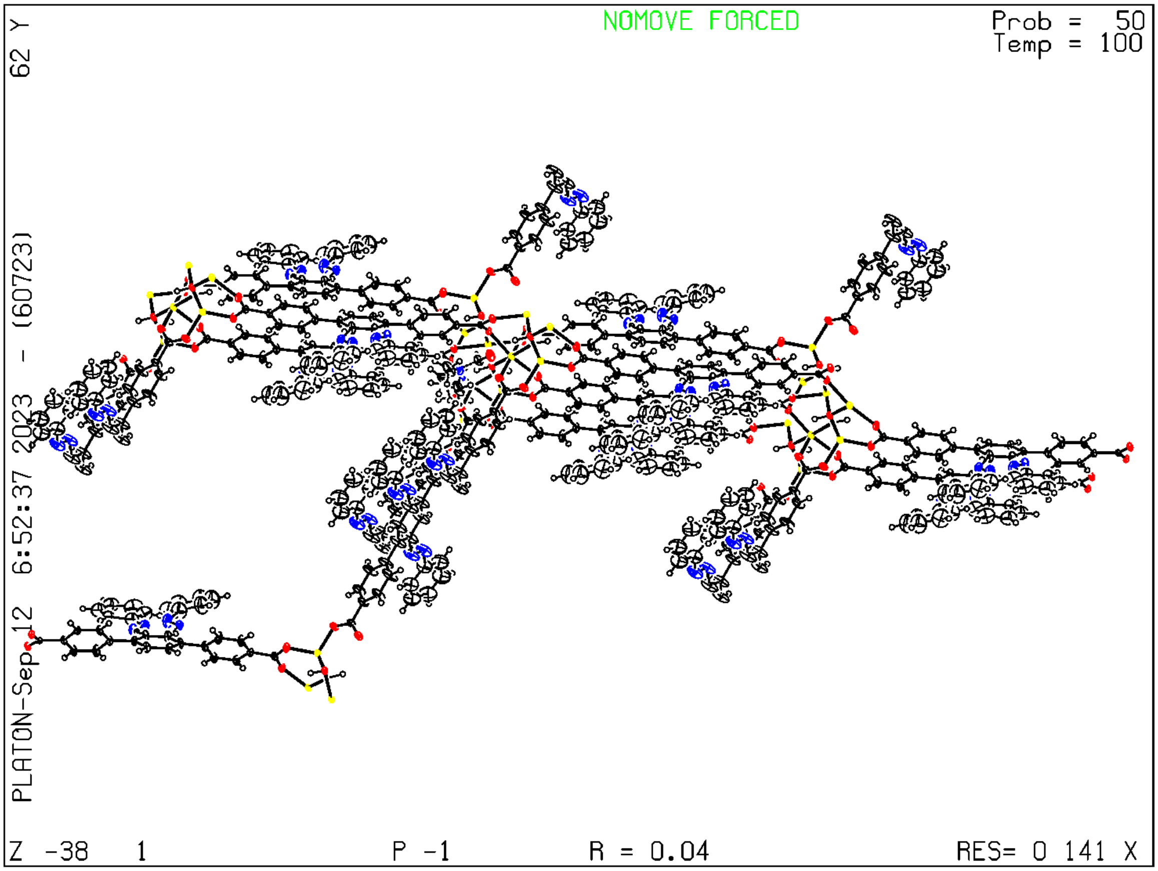

Supplement: Supplementary file 3 — Supporting File 3: advs75122‐sup‐0003‐DataFile.zip. [file ADVS-13-e24174-s003.zip › Data S3 (Azo-MOF cifreport).pdf]
